# Obtaining and Characterizing Poly(Acid Acrylic–co-Acrylamide) Hydrogels Reinforced with Cellulose Nanocrystals from *Acacia farnesiana* L. Willd (Huizache)

**DOI:** 10.3390/gels11020144

**Published:** 2025-02-18

**Authors:** Alejandra B. Navarro-Hermosillo, Gabriel Landázuri-Gómez, J. Félix Armando Soltero-Martínez, Manuel Alberto Gallardo-Sánchez, Jorge Alberto Cortes-Ortega, Carmen López-López, J. Jesus Vargas-Radillo, José Guillermo Torres-Rendón, Gonzalo Canché-Escamilla, Salvador García-Enriquez, Emma Rebeca Macias-Balleza

**Affiliations:** 1 Department of Chemical Engineering, University of Guadalajara, Guadalajara 44430, Mexico; alenicenavarro@gmail.com (A.B.N.-H.); gabriel.landazuri@academicos.udg.mx (G.L.-G.); j.soltero@academicos.udg.mx (J.F.A.S.-M.); 2 Department of Civil Engineering and Topography, University of Guadalajara, Guadalajara 44430, Mexico; manuel.gallardo@academicos.udg.mx; 3 Department of Chemistry, University of Guadalajara, Guadalajara 44430, Mexico; jorge.cortega@academicos.udg.mx; 4 Department of Wood Cellulose and Paper, University of Guadalajara, Guadalajara 44430, Mexicoj.vargas@academicos.udg.mx (J.J.V.-R.); jose.torres@academicos.udg.mx (J.G.T.-R.); 5 Material Unit, Yucatan Center for Scientific Research (CICY), Mérida 97205, Mexico; gcanche@cicy.mx

**Keywords:** acrylic hydrogels, cellulose nanocrystals, huizache

## Abstract

In this work, cellulose nanocrystals (CNCs) were obtained from the wood of *Acacia farnesiana* L. Willd (Huizache) via acid hydrolysis; then, they were used to reinforce polyacrylic acid–co-acrylamide (AAc/AAm) hydrogels synthesized in a solution process via in situ free radical photopolymerization. The nanomaterials were characterized using atomic force microscopy, dynamic light scattering (DLS), and the residual charge on the CNCs; the nanohydrogels were characterized using infrared spectroscopy, scanning electron microscopy, swelling kinetics, and Young’s modulus. Soluble-grade cellulose presented 94.6% α-cellulose, 0.5% β-cellulose, and 2.7% γ-cellulose, as well as a viscosity of 8.25 cp and a degree of polymerization (DP) of 706. The CNCs averaged 180 nm in length and 20 nm in width. In the nanohydrogels, it was observed that the swelling kinetic behavior followed the Schott kinetic model, at times lower than 500 h; after that, it became linear. The results show that the hydrogel swelling capacity depended on the crosslinking agent and CNC concentration, as well as the CNC chemical and morphological properties, rather than the CNC source. The hydrogels with CNCs exhibited a decreased swelling degree compared to the hydrogels without CNCs. Young’s modulus increased with CNC presence and depended on the concentration and characteristics of the CNC as a crosslinking agent.

## 1. Introduction

Hydrogels are polymeric networks with a three-dimensional configuration capable of absorbing large amounts of water or biological solution due to the presence of hydrophilic groups, such as –OH, –COOH, –CONH_2_, and –SO_3_H, while maintaining structural integrity [1]. Due to the ability to mimic many of the physical properties of tissues, hydrogels are good candidates for multiple biomedical applications, such as cell culture substrates, cell encapsulation, drug delivery, optical and ophthalmological applications, human tissue, and duct prosthetics, as well as suture coatings and waste treatment [2]. Although most synthetic hydrogels are similar to biological tissue, they are generally fragile and prone to fracture at low strains, which makes their application difficult when high stress is required [3].

Many strategies have been proposed, including the synthesis of hydrogels using photosensitive initiators; this is a polymerization technique that is usually carried out in a short time, can be carried out at room temperature, does not require organic solvents, and offers dimensional control advantages [4]. Above all, it allows for the incorporation of additives or reinforcing agents such as cellulose, which is the most abundant organic substance on planet Earth, as it integrates most of the biomass.

The large quantities of agro-industrial waste in Mexico have sparked researchers’ interest in exploring alternative ways to use it [5]. *Acacia farnesiana* L. Willd, known as Huizache, is a plant native to tropical America, and it is found from the southern United States to Brazil, Colombia, and Peru. It is popularly called Mimosa farnesiana or white thorn. It is a species with clear potential for future value [6], and it is used for ornamental purposes [7,8]. The leaves, flowers, and fruits are used as fodder for cattle and goats [9,10,11]. The essential oil of the flowers is used as a flavoring for its pleasant violet aroma. The bark and pods are rich in tannins, so they are used to tan and dye leather [12]. The wood of this species is stiff, thus it is used to construct fences and tool handles. It has a high calorific value as a fuel (wood and coal) [13]. Recently, the seeds have been studied as a source for the production of biofuels [14], the obtaining of soluble-grade cellulose [15], the isolation of cellulose nanocrystals [16], and the phytoremediation of soils contaminated with crude oil [17]. In the literature, it is possible to find a large number of investigations about the isolation and production of α-cellulose and its transformation into nanocrystals; however, in the literature consulted in this study, we only found the report made by our working group [16]. One of the main advantages of using *Acacia farnesiana* as a CNC source is that the plant has a shorter growth cycle than traditional sources, is highly drought-tolerant, and thrives in arid or semi-arid regions, making it a sustainable and renewable cellulose source. There is little information about the use of wild lignocellulosic materials, such as Huizache, which, in many cases, is underutilized. The potential use of its biomass to generate cellulose nanocrystals could expand the range of opportunities for using this plant species among the various nanotechnology applications, adding more value to this biomaterial.

Through different mechanical, chemical, enzymatic, and biological processes, it is possible to obtain cellulose nanofibers and nanocrystals (CNCs), which are the most basic structural forms of this polysaccharide and provide considerable improvements to the mechanical properties of the material in which they are present [18,19,20,21,22]. In the 1940s, Rånby first reported that colloidal suspensions of cellulose can be obtained via the controlled degradation of cellulose fibers catalyzed by sulfuric acid [23,24]. CNCs are a promising renewable nanomaterial with unique properties, such as high strength, liquid crystalline behavior, lightweight, biodegradability, and general biocompatibility [25,26]. CNCs obtained from various sources via acid hydrolysis have unique characteristics that make them suitable for multiple applications. The yield of CNCs obtained via acid hydrolysis, derived from *Pennisetum purpureum* [27], eucalyptus Kraft pulp [28], cotton fibers [29], and agave bagasse [30], was reported to range from 1 to 70%, with sizes ranging from 120 to 220 nm and a sulfur content between 3 and 10 mg/g. Bleached pulp fibers were used to produce carboxylated CNCs and CNFs using maleic acid, thereby enhancing sustainability [31]. Hydrogels have been reinforced with cellulose nanofibers obtained from various botanical sources, such as Cypress [32], Radiata pine [33], Jute fiber [34], birch [35], fique tow [36] rice and oat husks [37], abacá [38], *Agave tequilana* [39], sugar cane bagasse [40], red oak and yellow poplar [41], and pineapple leaves [42]. The use of bacterial cellulose to obtain hydrogels has also been reported [43,44,45,46,47,48]. Notably, the use of CNCs has been reported to improve the mechanical properties of hydrogels for use in medical applications, such as the controlled release of drugs and tissue scaffolds [49]. CNCs obtained from commercial cotton were used in collagen hydrogels and improved the mechanical properties without altering their triple-helix structure [50]. CNCs obtained from commercial cellulose microcrystalline were modified with hydrophobic groups to improve interfacial compatibility and mechanical properties [51].

These biomaterials are obtained from the acid hydrolysis of wood fiber and plants. They also have excellent properties, are light and flexible, and are obtained from renewable sources, in addition to being low-cost [52]. These characteristics make nanocrystalline cellulose an interesting option for reinforcing polymeric matrices.

The production of cellulose nanomaterials from lignocellulosic biomass opens an opportunity to develop and apply new materials in nanotechnology [53]. In recent years, cellulose nanomaterial-based hydrogels have emerged as promising materials in the field of biomedical applications due to their low toxicity, biocompatibility, biodegradability, and excellent mechanical stability [54,55,56]. CNCs have been incorporated as fillers to reinforce polymeric hydrogels based on poly(vinyl alcohol) [57,58,59], poly(vinyl alcohol)/carboxymethylcellulose [60], α-cyclodextrin [61], polyethylene glycol [62], poly(N-isopropylacrylamide [63,64,65], polyacrylamide [66,67,68], poly(acrylic acid/acrylamide) [69], polyacrylic acid [70], sodium alginate-g-poly(acrylic acid-co-acrylamide) [71], pectin–polyacrylic acid [72], carboxymethylcellulose/hydroxyethylcellulose [73], carboxymethyl cellulose [74], gelatin [75], gelatin/alginate [76], sodium alginate/acrylamide [77], alginate/collagen [78], calcium alginate [79,80], starch [81], pectin [82], collagen [83,84], and chitosan [85,86]. Furthermore, CNC self-association and crosslinking in hydrogels are promoted by CaCl_2_ [87]. In all these systems, improved mechanical properties have been observed, even at low CNC loadings (generally less than 3 wt. %).

The objective of this work was to obtain cellulose nanocrystals from Huizache (*Acacia farnesiana* L. Willd) and apply them as reinforcements in poly(acrylic acid–co-acrylamide) hydrogels (AAc/AAm). We characterized them using infrared spectroscopy, swelling kinetics in water, scanning electron microscopy, and rheological properties. Moreover, the results were compared with those obtained using CNCs from agave bagasse (*Agave tequilana* Weber var. azul) and commercial CNCs from wood.

## 2. Results and Discussion

### 2.1. Soluble-Grade Pulp Properties

After hydrolysis and the alkali sulfite–anthraquinone–methanol (ASAM) process, the pulp was obtained with a yield of 42% and a Kappa number of 12. In the bleaching process, around 9% of the pulp was lost after cooking, leaving a yield of approximately 33% of soluble-grade cellulose from Huizache wood. Lopez (2012) reported yields of 50.1% for a soda process, 47.0% for a Kraft process, and 45.3% for a sulfite process for Huizache wood [88].

Other authors have reported similar values using hardwoods [89,90] or corn stalks [91]. Dissolving-grade pulp has some unique properties and characteristics, including a very high alpha-cellulose content (greater than 90%), a low hemicellulose content (3 to 6%), and traces of lignin and other impurities [92]. The key quality parameters of dissolving-grade pulp include alpha-cellulose content, alkali solubility, the degree of polymerization, molecular weight distribution, and reactivity [93]. The soluble-grade cellulose presented 94.6% α-cellulose, 0.5% β-cellulose, and 2.7% γ-cellulose, with 91% whiteness, as well as a viscosity of 8.25 cp, a degree of polymerization (DP) of 706, and a molecular weight of 241.452 g/mol. Cellulose materials have DPs that vary depending on the source, production process, and treatment [94]. DP values range from 100 to 3000 for commercial celluloses, with a value of 20,000 for cotton fiber secondary walls [95].

### 2.2. Cellulose Nanocrystal Characteristics

Table 1 shows the average results of the CNC hydrodynamic diameter obtained using DLS and the length obtained using AFM, as well as the residual load of acid groups per kg of CNCs. 

According to the DLS results, the size distribution of the CNC Hu-A type exhibited a monomodal pattern of the Gaussian type. In contrast, the size distribution of the CNC Hu-B, Hu-C, and Hu-D types was bimodal, indicating two CNC size populations, attributed to fines produced in the bleaching process or the hydrolysis conditions, as reported in our previous work [16].

**Figure 1:**
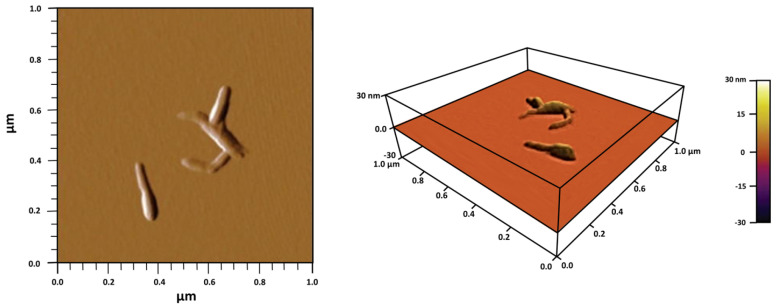
Image of CNCs obtained via hydrolysis of α–cellulose from Huizache with sulfuric acid, showing the amplitude and 3D projection technique.

The CNCs were analyzed using an atomic force microscope. It was found that the CNC Hu-A samples were homogeneous, with an average length of 180 ± 20 nm, a width of 20 ± 2 nm, a height of 7 ± 1 nm, and low dispersion in the size distribution. The CNC Hu-C and Hu-D samples showed two size populations, as reported by Ramirez et al., 2019 [16], while the width and height showed the same average values independent of the hydrolysis conditions. The dimensions of the CNCs obtained from wood cellulose were within the range mentioned by other authors [96,97]. In the images obtained from the morphological study of the nanocrystals, CNCs with irregular shapes could be observed (Figure 1).

### 2.3. Hydrogel Characterization

The AAc/AAm hydrogels synthesized after washing presented a reaction yield of 90 ± 2. Before washing, they were flexible; after washing and drying, they were transparent and hard.

#### 2.3.1. FTIR Spectroscopy of Hydrogels

**Figure 2:**
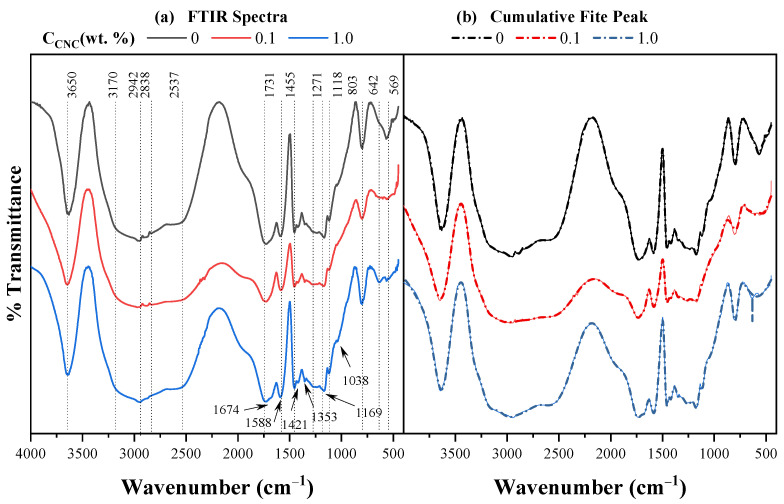
(**a**) FTIR spectra of AAc/AAm hydrogels with 0.5 wt. % NMBA and different concentrations of Hu-ACNCs (0, 0.1, and 1 wt. %). (**b**) Cumulative fit of the spectra in Figure 2a.

Figure 2a shows the FTIR spectra of the AAc/AAm (50/50) hydrogels crosslinked with n-methylen bis(acrylamide) (NMBA) 0.5 wt. % and photoinitiated (a) without cellulose (control sample), (b) with Hu-A CNC 0.1 wt. %, and (c) with Hu-A CNC 1 wt. %. Peaks corresponding to acrylic acid (AAc) can be observed. The band at approximately 3300 cm^−1^ is attributed to the OH groups [98]. The carboxyl group is observed at 1731 cm^−1^ and C-O is located at 1169 cm^−1^, while the C-O and O-H combinations are distinguished at 1422 and 1271 cm^−1^ [4]. Acrylamide (AAm) exhibits a combination of the stretching and flexion vibrations of N-H and C-N at 1353 and 642 cm^−1^, with the latter having a very low intensity. The two characteristic bands of amides can be observed, namely, Band I, located at 1688 cm^−1^, and Band II, located at 1588 cm^−1^; these are attributed to carbonyl stretching and the bending vibrations of the N-H bond, respectively, reflecting the presence of amide groups [99,100]. Bands I and II mask the band of the combination of the deformation and stretching vibrations of N-H and C-N at 1615 cm^−1^. The bands of CH_2_ are located at 2943 and 2883 cm^−1^ [4,101]. The stretching vibration bands of N-H for NH_2_ are associated with 3342 and 3200 cm^−1^. The crosslinking agent, NMBA, which has the same functional groups as AAm, exhibits bands at the same wavelengths [101,102]. However, cellulose exhibits bands attributed to the crystalline structure between 1420 and 1428 cm^−1^ and to the amorphous structure near 900 cm^−1^ [103]. Asymmetric and symmetric C-H vibration bands are located at 2942 and 2883 cm^−1^, respectively, and they overlap with those presented by the hydrogel components. The bands at 1038 cm^−1^, at 1169, and between 3600 and 3200 correspond to the C-O, C-O-C, and OH bonds, respectively [104,105,106]. It is also possible to observe characteristic signals of the AAc/AAm hydrogel, such as the band that appears around 3450 cm^−1^, which, according to Orozco-Guareño et al. (2011), is caused by the overlap of the absorption bands of the vibration of the O-H and N-H bonds corresponding to acrylic acid and acrylamide [107]. Asymmetric stretches of the COO-group are also found at 1455 cm^−1^. The band at approximately 2182 cm^−1^ corresponds to the presence of the C-N bond of AAm and NMBA.

In all cases, the bands observed for cellulose and the crosslinking agent are overlapped by the bands presented by the AAc/AAm hydrogel, making it impossible to determine the interactions of the cellulose and crosslinking agent in the hydrogel.

As the FTIR spectra of all hydrogels are similar, whether as a function of NMBA concentration, cellulose concentration, or CNC type, all samples exhibit the same bands at approximately the same wavelengths; qualitatively, no difference could be observed between them that could be attributed to the crosslinking agent or the cellulose, so a more in-depth analysis was carried out.

**Figure A1:**
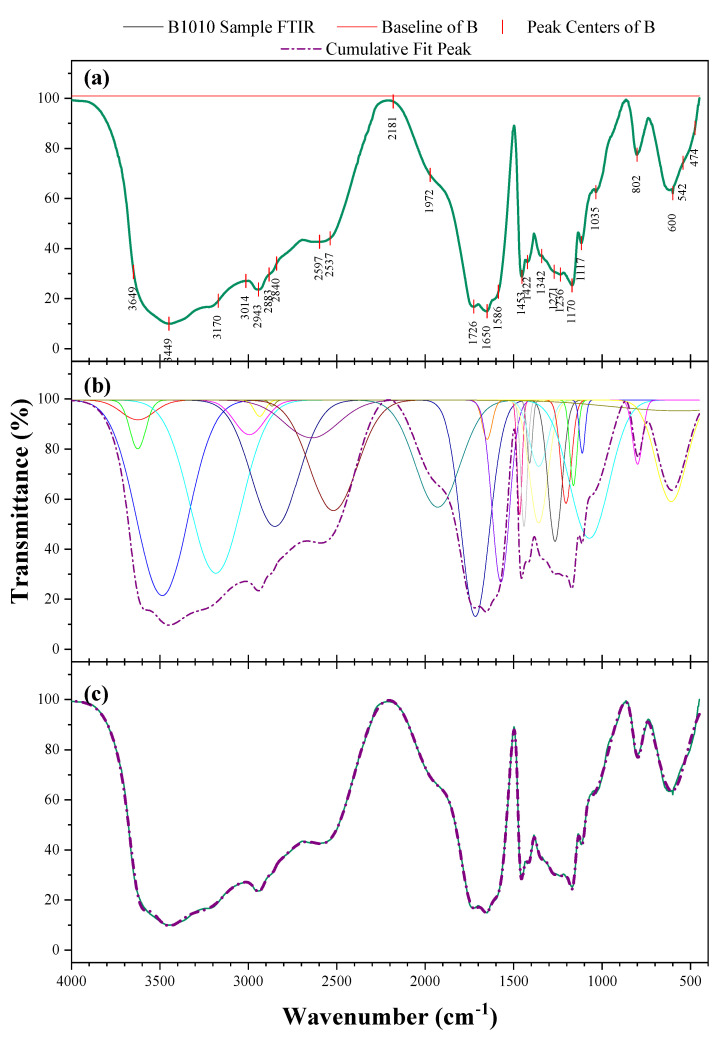
(**a**) FTIR for hydrogel with the Hu-B-type CNC with 1 wt. % NMBA and 1 wt. % CNC. The signaled peaks correspond to peaks for which the area was calculated. (**b**) The deconvoluted peaks obtained from the Gaussian deconvolution in Figure A1 (**a**) and the corresponding cumulative fit, each colored peak corresponds to the deconvoluted peak signaled in A1(a) at the same wavenumber as the center peak. (**c**) Comparison between the original FTIR.

The areas of all peaks in all spectra were determined via peak deconvolution using Origin software, Version 2021 (OriginLab Corporation, Northampton, MA, USA). A total of 28 peaks per spectrum were found using the local maximum method of peak detection. As an example, Appendix A Figure A1 shows the FTIR spectra analysis of the hydrogel with 1 wt. % NMBA and 1 wt. % Hu-B CNC, the wavenumber of each peak signaled in the original spectra as the deconvolution of the FTIR spectra peaks, and the cumulative fit (R^2^ = 0.999). The Gaussian function used to obtain the deconvolution of the spectra peaks is as follows:

(1) $$y=y_{0}+\frac{A}{w\sqrt{\frac{\pi }{2}}}e^{-2\frac{{\left(x-{\;x}_{c}\right)}^{2}}{w}}$$

where x and y are the wavenumber and transmittance, y_0_ is the offset, and x_c_, w, and A correspond to the peak center, width, and area, respectively. The results obtained from the analysis are shown in Appendix A, Table A1, with the area percent average and variation in each peak as the functional group associated with the wavenumber for the control and composite samples.

**Figure 3:**
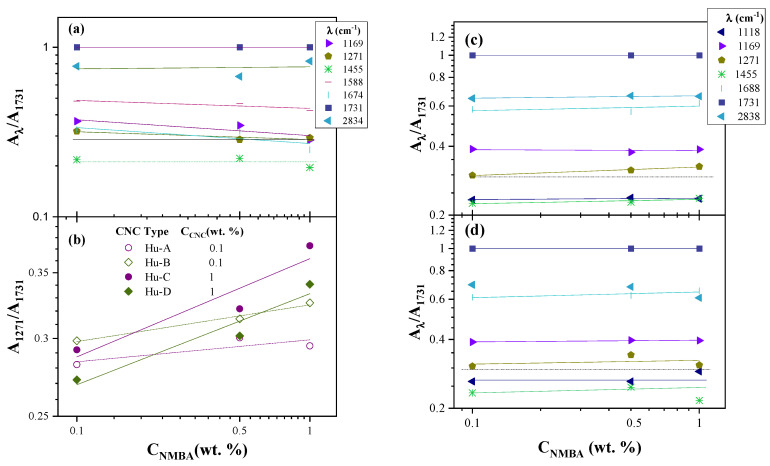
A_λ_/A_1731_ area ratio for FTIR spectra of AAc/AAm hydrogels as a function of NMBA concentration (**a**) for control samples; (**b**) A_1271_/A_1731_ for hydrogels containing different types and concentrations of CNCs; and (**c**,**d**) A_λ_/A_1731_ for hydrogels containing CNC 0.1 and 1 wt. %, respectively. The lines correspond to data power law fit.

The cumulative fit of the spectra in Figure 2a is shown in Figure 2b, and it can be observed that the approximation is very precise. The results indicate that, for the control samples (the hydrogels without cellulose, with NMBA 0.1, 0.5, and 1 wt. %), the percentage of variation in the areas of the peak located at 1731 cm^−1^ is lower than 1% (0.6%, see Table A1); this corresponds to the carboxyl group of AAc, AAm, and NMBA and it can be considered constant for all formulations, as the monomer composition is constant. Thus, the peak areas of all FTIR spectra are normalized with respect to the peak area at 1731 cm^−1^ (A_*λ*_/A_1731_); this standardization has also been performed for the FTIR of acrylic hydrogels [4]. The A_*λ*_/A_1731_ area ratio as a function of the NMBA concentration for the control samples is shown in Figure 3a for various wavelengths (1169, 1271, 1455, 1588, 1688, 1731, and 2838 cm^−1^). It can be observed that the area ratio decreases slightly for most of the peaks at the different wavelengths according to the power law ($\frac{A_{\lambda }}{A_{1731}}=a{\left[NMBA\right]}^{b}$). Still, this ratio shows no dependence on the NMBA concentration for the wavelengths of 1455 and 2838 cm^−1^ (CH_2_ symmetric deformation and stretching vibrations, respectively), indicating that the bands at these wavelengths are independent of the NMBA concentration. Upon the addition of cellulose, the A_*λ*_/A_1731_ area ratio of most of the peaks remains constant, as can be seen in Figure 3c,d, which show the A_*λ*_/A_1731_ area ratio for the AAc/AAm hydrogels prepared with Hu-B CNC 0.1 and 1 wt. %, respectively. The symbols represent the experimental data, and the lines represent the power fit ($\frac{A_{\lambda }}{A_{1731}}=a{\left[NMBA\right]}^{b}$) of these values. These results show that the only A_*λ*_/A_1731_ area ratio dependent on the NMBA concentration is for *λ* = 1271 cm^−1^ (OH deformation vibration of AAc), where a small increase with the NMBA concentration is observed (for a better appreciation, a dotted horizontal line is placed as a reference). These results are also observed for the Hu-B, Hu-C, and Hu-D cellulose types with CNC 0.1 and 1 wt. %. Figure 3b shows the A_1271_/A_1731_ area ratio for the four cellulose types, with CNC 0.1 wt. % (Hu-A and Hu-B types) and CNC 1 wt. % (Hu-C and Hu-D types) compared to the control samples; it can be seen that the A_1271_/A_1731_ area ratio decreases with an increasing NMBA concentration and increases when cellulose is added, with this increase being more drastic with CNC 1 wt. % than with CNC 0.1 wt. %. Thus, according to the results shown in Figure 3, the bands at 1455 and 2838 cm^−1^ are independent of the NMBA concentration for the hydrogels without cellulose, and the band at 1271 cm^−1^ is simultaneously dependent on the NMBA concentration for the hydrogels with cellulose, indicating a chemical interaction between cellulose and the AAc/AAm hydrogel, as the area ratios decrease with the NMBA concentration for the control samples and increase when CNCs are added.

**Figure 4:**
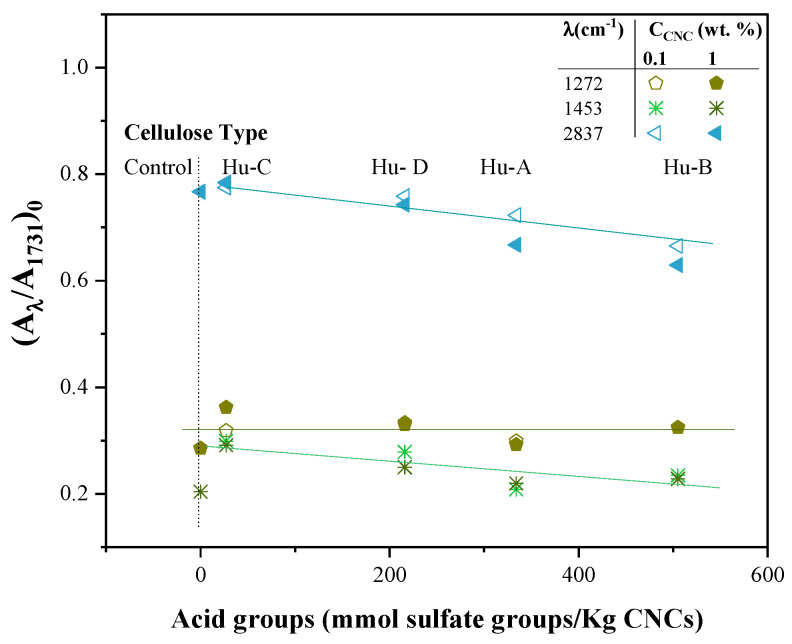
(A_λ_/A_1731_)_0_ as a function of the C_AG_ in CNCs for λ = 1271, 1455, and 2838 cm^−1^ and for the different CNC types. The lines are visual aids.

To relate these results to the chemical composition of the CNCs, the value of the ordinate at the origin was taken from the power fit regressions in Figure 3b,c for the CNC Hu-A type, (A_*λ*_/A_1731_)_0_, and the same was carried out for the other types of CNCs. The (A_*λ*_/A_1731_)_0_ values for λ = 1271, 1455, and 1834 cm^−1^, as a function of the NMBA concentration for each type of CNC, were graphed as a function of the concentration of the acid groups in the nanocrystal (taken from Table 1) and are shown in Figure 4; here, it could be observed that, for wavelengths of 1455 and 2838 cm^−1^, (A_*λ*_/A_1731_) decreases with the increase in the degree of cellulose sulfation, while for 1271 cm^−1^, independence from the degree of sulfation was observed. By analyzing each band, it was found that the interactions were due to the combination of the C-O and O-H bonds at 1271 cm^−1^ of AAc and dependent on the NMBA concentration (Figure 3b–d); it has been reported that the carboxylic groups of the copolymer have complex electrostatic interactions with the cationic groups of acrylic acid during polymerization [108]. While the bands at 1455 and 2838 cm^−1^, affected by the presence of CNCs and mainly attributed to the symmetrical stress and strain vibrations of CH_2_, respectively [98], decreased with increasing concentrations of sulfate groups, the band at 1455 also overlapped with the asymmetric stretches of the COO-group. Then, the chemical interaction between cellulose and the hydrogel decreased as the degree of sulfation increased; i.e., there was a decrease in the number of OH groups on carbon 6 of cellulose. The above suggests that a chemical interaction took place between the OH groups of cellulose and the acidic monomer. It has been reported that acrylic acid and cellulose exhibit chemical interactions at 1651, 1450, and 1170 cm^−1^ [109].

#### 2.3.2. Hydrogel Swelling Kinetics

**Figure 5:**
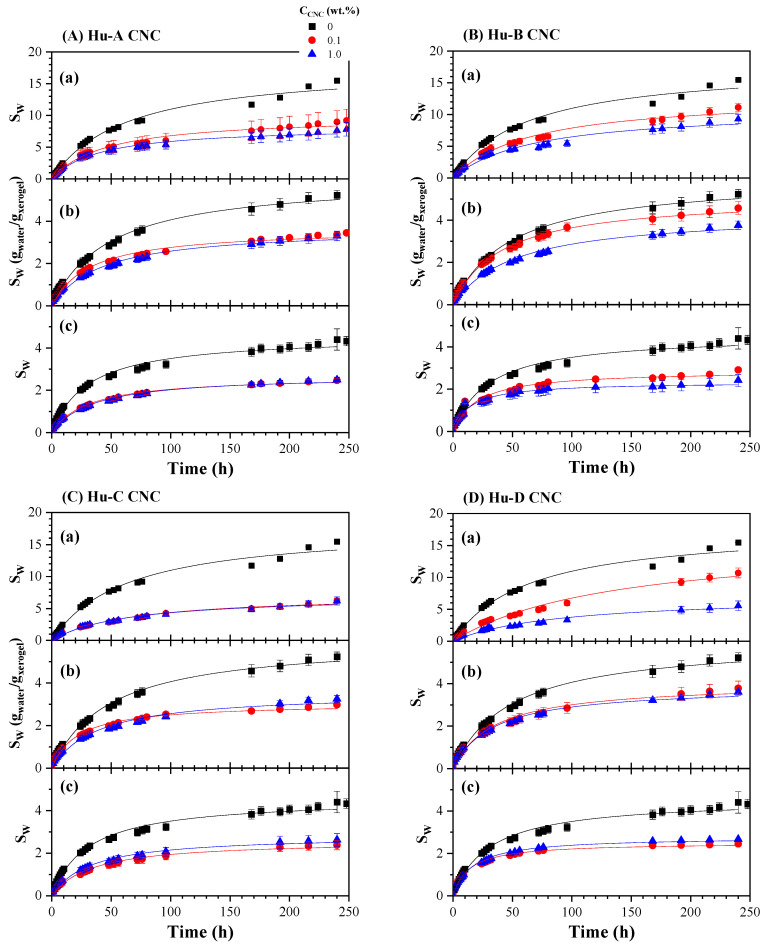
Swelling kinetics of AAc/AAm hydrogels with different CNC types ((**A**) Hu-A, (**B**) Hu-B, (**C**) Hu-C, and (**D**) Hu-D) and concentrations (0, 0.1, and 1 wt. %) and different NMBA concentrations ((**a**) 0.1, (**b**) 0.5, and (**c**) 1 wt. %).

The water remained colorless, odorless, and visually slime-free during hydrogel swelling kinetics. The hydrogels were placed in 100 cm^3^ containers filled to 80% of their capacity so that they were always submerged in double-distilled water. The containers were kept closed to prevent water evaporation and swelling system contamination. The effect of the NMBA concentration (0.1, 0.5, and 1 wt. %) on the swelling kinetics of the AAm/AAc (50/50) hydrogels as a function of the CNC concentration (0.1 and 1 wt. %) obtained from Huizache under different hydrolysis conditions (Hu-A, Hu-B, Hu-C, and Hu-D treatments) can be observed in Figure 5A, Figure 5B, Figure 5C and Figure 5D, respectively. The symbols represent the experimental data, while the lines correspond to the second-order kinetics model proposed by Schott [110], the parameters of which are reported in Table 2. The experimental data and the Schott equation parameters are the average of five determinations. A statistical analysis of the hydrogel swelling kinetics can be observed in Table 3, which shows that the effects of the NMBA concentration and CNC type are significantly different, and the interactions between the NMBA concentration and CNC concentration and type are significant. Figure 5A shows the swelling kinetics as a function of the Hu-A-type CNC content and NMBA concentration: (a) 0.1, (b) 0.5, and (c) 1 wt. %. Most of the samples reached equilibrium within 100 h; however, the kinetics were followed for up to 10 days, and it was noted that the amount of water absorbed by the hydrogel decreased for both hydrogels with CNC 0.1% and 1% compared to the control sample (without CNC). This occurred for the three NMBA concentrations used (0.1, 0.5, and 1 wt. %) and is shown in Figure 5(Aa–Ac). This effect probably occurred because the crosslinking density increased with the concentration of CNC, as it acted as a crosslinking agent, hindering water absorption [54]. This phenomenon was also observed by Lim et al. (2017) when using cellulose nanocrystals as a reinforcing material in poly(acrylic acid)-based hydrogels with 5, 10, 15, 20, and 25% by weight of CNCs. Their results showed a higher degree of swelling in hydrogels with 5% *w/w* nanocrystalline cellulose, while the incorporation of more than 5% *w/w* tended to decrease the swelling ratio. They attributed this behavior to the increase in the number of hydrophilic hydroxyl groups, which facilitated the absorption of water within the hydrogel [111]. The decrease in swelling concerning the CNC content was dependent when C_NMBA_ was 0.1%; however, for C_NMBA_ 0.5 and 1 wt. %, no considerable changes were observed concerning the CNC content. In hydrogels based on sodium acrylate and N-acrylamide [111], 2-hydroxiethylmethacrylate [112], and NIPAM [113], increasing the crosslink concentration reduced the equilibrium degree of swelling.

The results obtained with the CNC Hu-D type are similar to those obtained with the Hu-A-type cellulose (Figure 5D). In contrast, with the Hu-C cellulose type (Figure 5C), there are no considerable changes with respect to the cellulose content for the three NMBA concentrations; even with 0.5 and 1 wt. % NMBA, there is a lower degree of swelling with 0.1% CNC than with 1% CNC (Figure 5(Cb,Cc)). However, for the Hu-B-type cellulose, shown in Figure 5B, the hydrogel swelling gradually decreases with the cellulose concentration for the three NMBA concentrations. These behaviors can potentially be attributed to the degree of cellulose sulfation. By analyzing the two extremes, the Hu-B and Hu-C samples, whose acid group concentrations are 505 and 27 mmol/Kg cellulose, respectively, it is noted that, when the acid group concentrations are low, the interactions between the CNCs and hydrogel are greater than when the acid group concentrations are high (see Figure 4). In other words, the more significant the degree of crosslinking and the lower the degree of sulfation, the lower the degree of hydration of the hydrogel; that is why the hydrogels synthesized with the Hu-C-type CNC (Figure 5C) present a lower degree of hydration, and this is independent of the cellulose concentration. Nevertheless, the hydrogels synthesized with the Hu-B-type CNC (Figure 5B), with a high degree of sulfation and, therefore, a low level of crosslinking, exhibit a degree of hydration dependent on the CNC concentration. The hydrogels with Hu-A and Hu-C, whose acid group concentrations are 334 and 216, respectively, only exhibit concentration dependence at low NMBA concentrations (Figure 5A,C). The acid groups influence and enhance the swelling kinetics because of the interplay between osmotic pressure and ionic interactions [114], and a decrease in pH reduces swelling due to the acidic groups transforming into undissociated forms [114,115].

**Figure 6:**
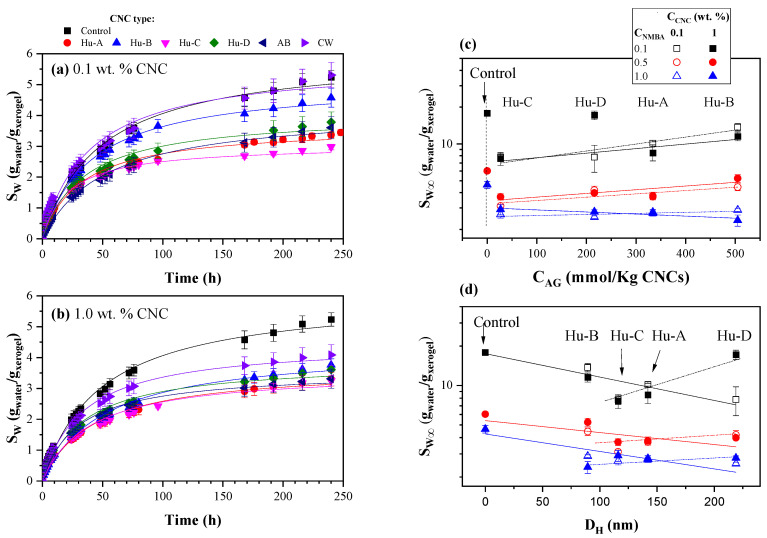
Effect of CNC type on swelling kinetics with CNC (**a**) 0.1 and (**b**) 1 wt. % and NMBA 0.5 wt. %. Dependence of S_W∞_ on (**c**) acid group concentration in cellulose and (**d**) CNC hydrodynamic diameter. The lines are visual aids.

Table 2 shows the Schott equation parameters [110] for the AAc/Aam hydrogel swelling kinetics as a function of the CNC type and concentration and NMBA concentration; each value corresponds to an average of five experiment fits, and the fit for the experimental data average is similar to the average of five fits. Table 3 shows a statistical analysis of the data in Table 2. This table shows that the swelling capacity decreases with the NMBA concentration for the control sample and for the AAm/AAc hydrogels reinforced with CNC 0.1 and 1 wt. %; however, the K value increases with the CNC concentration only for NMBA 0.1 wt. %, and for NMBA 0.5 and 1 wt. % it decreases for most samples or remains at a similar valor, considering the standard deviation. As shown in Figure 5, SW∞ achieves a similar valor for 1 wt. % NMBA, independent of the Huizache CNC concentration (2.70 ± 0.18); for NMBA 0.5 wt. %, excluding Hu-B, the average value is 3.74 ± 0.36. The CNC type AB and CW values are higher than those obtained for Huizache. A comparison of the effects of different CNC sources on swelling kinetics is shown in Figure 6.

Figure 6 shows the swelling kinetics of the AAc/AAm hydrogels with 0.5% NMBA and different CNC concentrations ((a) 0.1 and (b) 1 wt. %) and CNC types (CNCs obtained from Huizache under different hydrolysis conditions (Hu-A to Hu-D), CNCs obtained from agave bagasse (AB), and commercial CNCs obtained from wood (CW)). In all cases, the degree of swelling, S_W_, was lower for the hydrogels with CNCs than for those without CNCs. However, differences were observed with respect to the CNC content: For 0.1 wt. % CNC (Figure 6a), the sample containing CW did not show any difference from the control sample. The other samples showed a lower degree of swelling, with the hydrogel with the CNC Hu-C type showing a lower degree of swelling, and the Hu-B sample showing a higher degree of swelling. When 1% CNC was added (Figure 6b), most hydrogels presented a swelling degree between 2.5 and 3, while the sample with CW exhibited a higher swelling degree (3.5). In both cases, the sample with CW exhibited a higher swelling degree than the samples in which Huizache or agave bagasse CNCs were used. This behavior may be attributed to the nanocrystal size, which was 4 to 7 times larger than in the other formulations.

However, the difference between all types of cellulose can be attributed to the degree of sulfation of the CNCs. Figure 6c shows the equilibrium swelling S_W∞_, which was obtained from the adjustments to the second-order Schott model [110], as a function of the acid group concentrations (from Table 1) for the different CNC types. It can be observed that, for NMBA concentrations of 0.1 and 0.5 wt. %, the degree of swelling increases with the degree of sulfation; i.e., the greater the swelling, the lower the degree of crosslinking. This suggests that, by increasing the concentration of sulfate groups, the concentration of OH on cellulose carbon 6 is reduced, as well as the number of interaction sites between the cellulose and hydrogel, which is also confirmed via FTIR. Conversely, with 1 wt. % NMBA, the lowest degrees of swelling are observed regardless of the amount of cellulose. For CNC 0.1 wt. %, S_W∞_ remains almost invariable with C_AG_, and with 1 wt. %, a decrease in the degree of swelling is observed. This behavior may indicate that CNC also acts as a crosslinking agent at these CNC concentrations. Finally, the effect of CNC size on the swelling degree is shown in Figure 6d, where S_W_ is also observed to depend on the CNC concentration. The swelling degree decreases with the nanocrystal length (denoted by the solid line), perhaps because once cellulose has interacted with the polymer, increasing the nanocrystal length increases the number of possible interactions between them. For CNC 1 wt. %, the swelling degree increases for sizes greater than 100 nm. Thus, there is a dependence between the hydrogel swelling degree and the CNC concentration and length.

**Figure 7:**
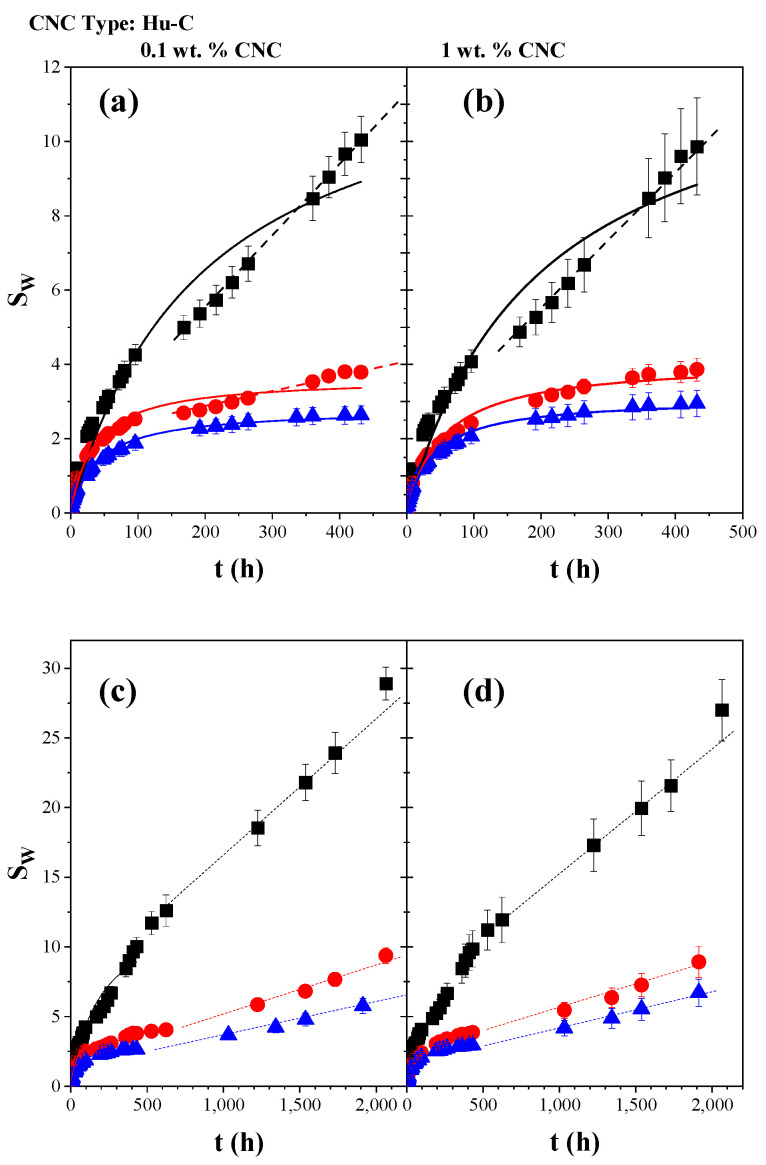
Swelling kinetics of hydrogels with the Hu-C-type CNC at 500 h with (**a**) 0.1 and (**b**) 1 wt. % CNC and at 2000 h with (**c**) 0.1 and (**d**) 1 wt. % CNC. The continuous lines correspond to the Schott model fit for times lower than 500 h, and the dashed lines correspond to the linear fit for times higher than 500 h.

According to Işık, in the case of acrylic acid-rich hydrogels, the swelling capacity is possibly controlled by the acrylic acid portion in the copolymer [116]. This behavior is probably attributed to the intermolecular hydrogen bonds between carboxylic acids and amide groups and the intramolecular hydrogen interactions between amide groups. Those results led us to infer that the nature of the crosslinking agent strongly impacts the hydrogels’ absorption properties, even though the monomers and their ratio are similar. In the AAc/AAm hydrogel reported in the present work, a pseudo-equilibrium was reached at approximately 100 h. As the hydrogels continued to absorb water very slowly, they were measured for a more extended period of 10 days, and the results are presented in Figure 5 and Figure 6. However, water uptake continued to increase slightly and was monitored for 2000 h. Figure 7 shows the swelling kinetics of the AAm/AAc hydrogels with 0.1, 0.5, and 1 wt. % NMBA at 500 h with (a) 0.1 and (b) 1 wt. % Hu-C-type CNC and at 2000 h with (c) 0.1 and (d) wt. % Hu-C-type CNC. The symbols represent the experimental data, and the lines correspond to the Schott model [110]. In Figure 7a,b, the samples with 0.1 and 1% cellulose reached a steady state at approximately 100 h; as can be seen in Figure 5, these steady states were not prolonged for the samples without CNC or for the sample with 0.1 wt. % NMBA and 0.1% CNC. After 240 h, there was a new increase in the swelling. Figure 7c,d show the swelling kinetics up to 2000 h. Evidently, after 500 h, the swelling increased linearly with time without reaching a new steady state. This behavior occurred in all hydrogels, both without cellulose and with cellulose, and for all NMBA concentrations. The lines correspond to a linear regression, and the slopes are shown in Table 4. This slope represents the hydrogel swelling rate at a significant or very long time, while all the lines start at the origin. A statistical analysis of the linear regression of the swelling kinetics at very long times is presented in Table 3, which shows that the interactions between the NMBA concentration and the CNC concentration and type were significant. At the same time, there was no significance between the CNC concentration and type.

Orozco-Guareño et al. (2011) established that different behaviors can be observed depending on the degree of crosslinking and the presence (or absence) of specific interactions between comonomers in the copolymer chain [107]. As shown in Table 2, the slope decreases with the NMBA and CNC concentrations, and the CNC characteristics play an important role in the swelling behavior.

#### 2.3.3. Hydrogel Morphology Determined via SEM

**Figure 8:**
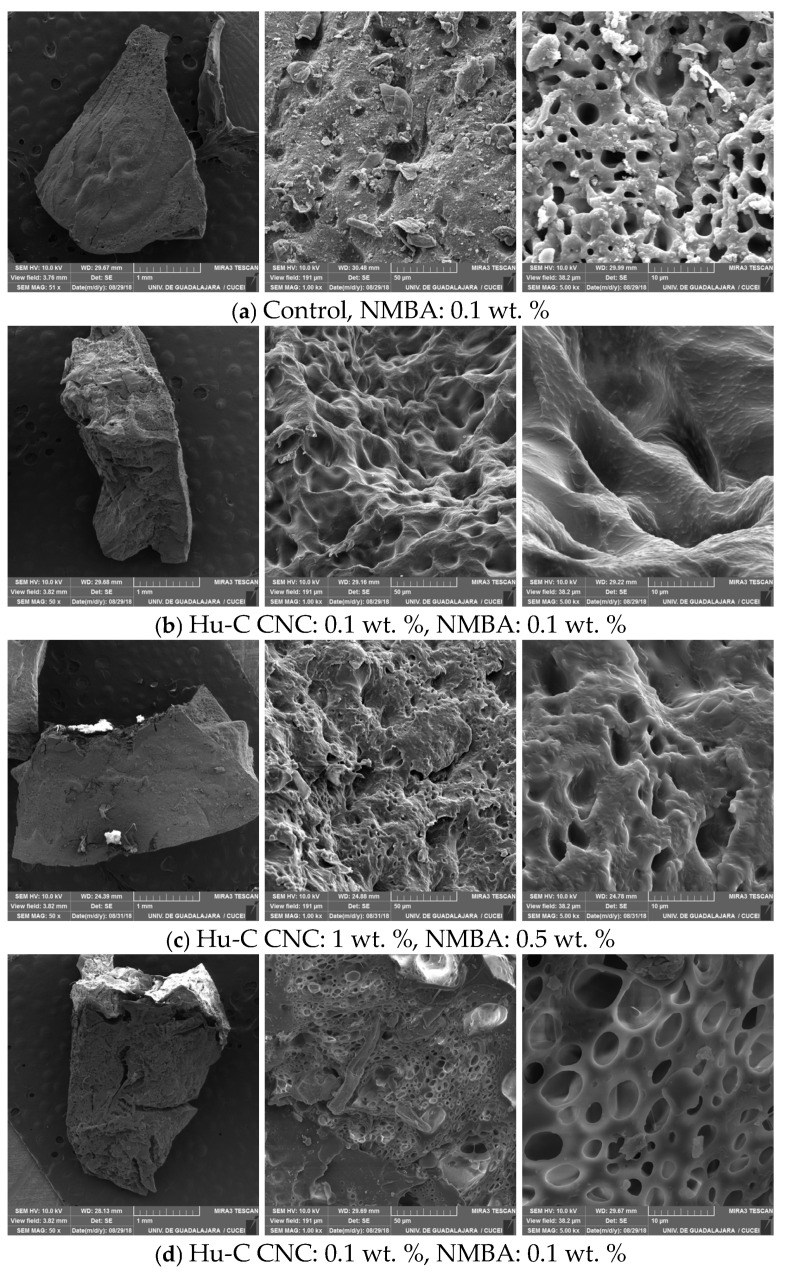
SEM images of AAc/AAM hydrogels: (**a**) control sample with 0.5 wt. % NMBA and 3 h of hydration; (**b**) sample with CNC 0.1 wt. %, NMBA 0.1 wt. %, and swelling time of 3 h; (**c**) sample with CNC 1 wt. % CNC, NMBA 0.5 wt. %, and swelling time of 3 h; and (**d**) sample with 0.1 wt. % CNC, 0.1 wt. % NMBA, and swelling time of 8 h.

Porosity in this type of material is essential, as it can determine the water and ion absorption capacities. Figure 8 shows micrographs of the hydrogels with different Hu-C and NMBA concentrations after 3 and 8 h or swelling time (Figure 3a–c and Figure 3d, respectively) at magnifications of 50x, 1kx, and 5kx. The images on the left (50x) show the hydrogels obtained once swollen, frozen, fractured, and freeze-dried; porous solids can be observed in all cases. Figure 8a (1kx magnification) shows an image of the control sample with 0.1 wt. % NMBA; pores between 5 and 20 μm can be observed, and the topology of the hydrogel exhibits tiny pores distributed in a non-uniform manner. When zooming in (Figure 8a, 5kx magnification), a non-homogenous distribution of pores can be observed, with sizes between 1 and 5 μm, and small protuberances are distributed randomly on the pore walls. Figure 8b (1kx magnification) presents images of the hydrogels with the Hu-C-type CNC (0.1 wt. %). A structured material with pores between approximately 10 and 20 μm can be distinguished when increasing the magnification to 5kx, and the hydrogel walls present elongated and thin bumps, giving a “chicken skin” appearance. By increasing the CNC and NMBA concentrations (Figure 8c, 1kx magnification), the pore size decreases (5–10 μm), which is attributed to a lower degree of swelling and a higher level of crosslinking. In Figure 8c, (5kx magnification), it can be seen that the walls of the hydrogel are not entirely smooth, and small protuberances are observed. Figure 8d shows micrographs of a hydrogel with a longer hydration time; the pores have a better definition, with a non-uniform size distribution, and the walls are smoother than those with a lower degree of swelling (Figure 8b). Photographs of the swollen hydrogels as a function of time for various CNC concentrations and types are shown in Appendix B as a visual reference.

Li et al. (2023) reported on the morphology of collagen hydrogels reinforced with vast quantities of CNCs (5–20 wt. %) compared to our research. At low CNC concentrations of 5 and 10 wt. %, they found interconnected porous structures with pores inside; at higher concentrations of 15–20 wt. %, the hydrogel was more compact, the porosity decreased, and the surface was smoother [50].

Wong et al. (2015) synthesized polyethylene oxide hydrogel films using various crosslinking agent concentrations [117]. They studied swelling kinetics and mechanical properties and presented a very detailed morphology analysis conducted using SEM. The images are similar to those obtained in our research (Figure 8a–c). They described hydrogel as a mesh structure with interconnected micropores, empathizing that equilibrium was achieved in only five hours because the channels could transport water. They determined that the mesh size diminished with the crosslinking concentrations, and the values decreased from 10.7 to 1.6 nm. Xion et al. (2014) reported the morphology of superabsorbent polymers as a function of the type or crosslinking agent, and the images exhibited a porous architecture with different pore sizes and distributions due to the crosslinking functional groups, distance between bonds, and steric effects [118]. Their images are similar to those in Figure 8c. Li et al. reported the morphology of collagen hydrogels reinforced with vast quantities of CNCs (5–20 wt. %) compared to our research. At low CNC concentrations of 5 and 10 wt. %, they found interconnected porous structures with pores inside; at higher concentrations of 15–20 wt. %, the hydrogel was more compact, the porosity decreased, and the surface was smoother [50]. 

For the hydrogels without CNCs, the pore size decreased with the NMBA concentration; at a lower NMBA concentration, the swelling ratio was higher, and the pore size was larger. The same occurred with the CNC concentration; CNC 0.1 wt. % resulted in a larger swelling degree than CNC 1 wt. % and a lower swelling degree than the hydrogels without CNC. According to Canal and Peppas (1989), the swelling ratio decreases with the pore size [119]. Furthermore, SEMS revealed that hydrogels with irregular structures exhibit swelling behavior dependent on the acrylic acid content, with the swelling ratio increasing with the acrylic acid concentration at neutral and alkaline pH values [120]. Rajabali, 2019, discussed the effect of hydrogel porosity on swelling capacity; as the porosity increases, the swelling ratio also increases [121]. Jin et al. (2009) used Fick’s Law to obtain the diffusion coefficient and demonstrated that the swelling mechanism of p(VP-co-MMA/NIPAM) semi-IPN hydrogels depends on the microstructure, with a larger pore size exhibiting a faster swelling rate [122]. Conversely, Wang et al. (2018) showed that the time and crosslinking process can be controlled to obtain hydrogels with different microstructures; at a low crosslinking capacity, the chains have greater mobility, leading to a larger water bed, while a denser network leads to a smaller water bed [123].

**Figure 9:**
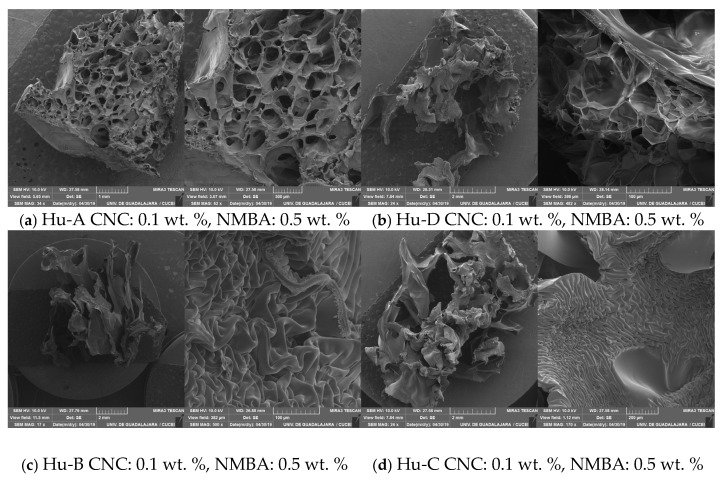
SEM images of AAc/AAM hydrogels with 0.1 wt. % CNC; 0.5 wt. % NMBA; and different CNC types, namely, (**a**) Hu-A, (**b**) Hu-D, (**c**) Hu-B and (**d**) Hu-C.

Figure 9 shows SEM images of the hydrogels swollen at equilibrium (72 h), or in our case, in a pseudo-steady state, with 0.5 wt. % NMBA and 0.1 wt. % CNC of the (a) Hu-A and (b) Hu-D types. In the images on the left, complete samples are shown, and, unlike the images obtained a few hours after swelling (Figure 8), the pores can be observed at 34 and 24x. In the close-up, it is evident that the walls of the pores are smoother than those observed at low swelling times (Figure 8); pores are observed between 100 and 500 μm in Figure 9a, and between 25 and 100 μm in Figure 9b. At very long hydration times, the hydrogels with 0.5 wt. % NMBA and 0.1 wt. % CNC of the (a) Hu-B and (b) Hu-C types, when freeze-dried, no longer have the appearance of a porous solid but instead look like long lamellae whose walls have smooth and rough areas randomly distributed (Figure 9c,d); when magnifying, the rough part’s curved, grooved textures can be observed.

#### 2.3.4. Hydrogel Rheological Characterization

**Figure 10:**
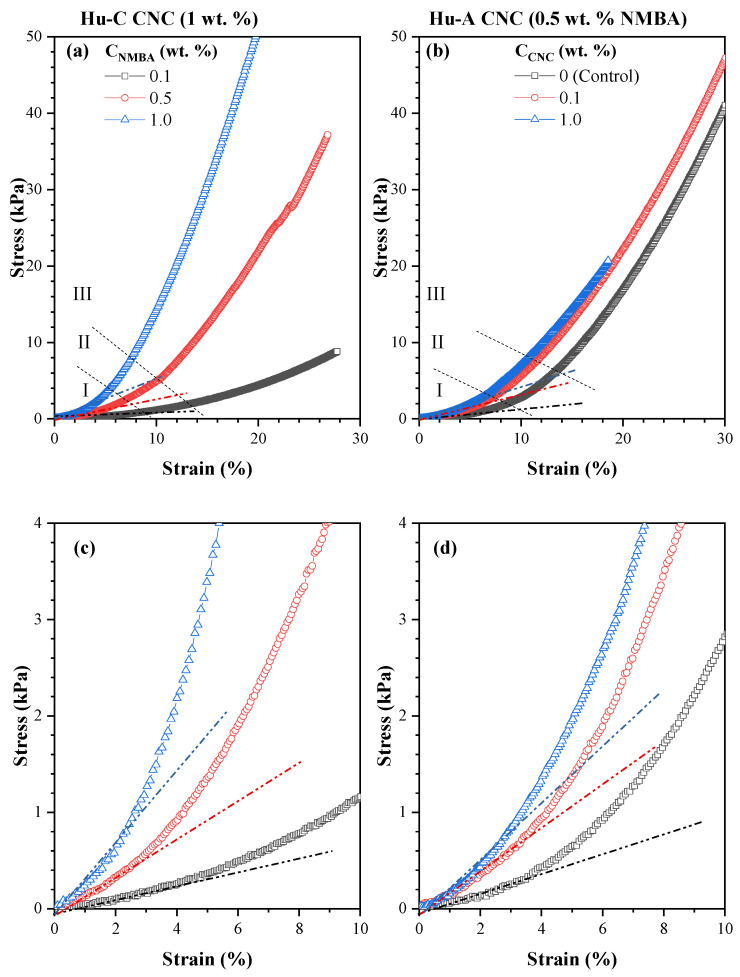
(**a**) Effect of NMBA concentration and (**b**) effect of CNC concentration on Young’s modulus using type C CNC, the dotted lines delimit the deformation zones. (**c**,**d**) Magnifications of lineal zone from (**a**) and (**b**), respectively, and the dashed lines correspond to linear fit of first linear region.

Young’s modulus and compression tests (squeeze) were performed to determine the hydrogels’ mechanical properties. Figure 10a,b show stress–strain curves (a) as a function of the NMBA concentration and (b) as a function of the CNC concentration, and Figure 10c,d show magnifications of Figure 10a and Figure 10b, respectively. In both rheograms, the curves consist of distinct zones [124]: The elastic region (I) is the initial zone where the material exhibits a linear relationship between the stress and strain, and the slope is known as Young’s modulus [124,125]; in this region, the material deforms elastically. It is able to return to its original shape once the load is removed, and this is better observed in Figure 10c,d. The second zone (II) is the yield point, and it marks the transition from elastic deformation to plastic deformation. Beyond this point, the material will not return to its original shape once the stress is removed [124]. Region III is the plastic region, where the hydrogel exhibits permanent deformation. The stress continues to increase, but the strain increases more rapidly, and the structure of the material changes due to the sliding of the polymer chains. At higher deformations, the ultimate strength (Zone IV) and the fracture point (Zone V) can be found, but they are not observed in this work. The ultimate strength is the maximum stress that the material withstands before failure, and this zone also marks the limit of the mechanical integrity of the hydrogel [126]; the fracture point is the zone where the material fails completely.

The effect of NMBA at a constant concentration of 1 wt. % Hu-C CNC after 300 h of swelling is shown in Figure 10a,c. It can be observed that a higher NMBA concentration results in an increase in the slope of the graph, i.e., in Young’s modulus. This result may be because the strength of the material increases dramatically with an increasing crosslinking density. It has been shown that adding more crosslinking agents can easily increase the swelling capacity. A wide range of studies have been carried out on the dependence of mechanical properties on the concentration of the crosslinking agent in hydrogels [127]. The effect of the CNC concentration on the equilibrium properties of the hydrogels is shown in Figure 10b. It can be observed that, as the CNC concentration in the hydrogel increases, Young’s modulus increases, indicating that the CNC acts as a reinforcing material. Both figures show that deformation in the lineal zone decreases with the NMBA and CNC concentrations.

**Figure 11:**
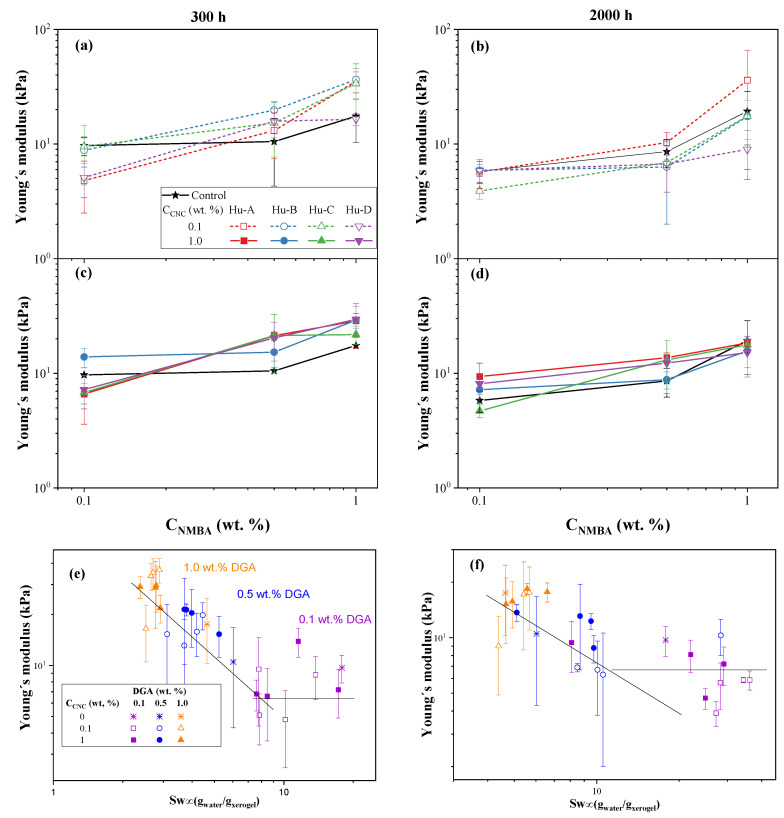
Young’s modulus of hydrogels with and without CNCs: as a function of the NMBA concentration for different CNC concentrations at hydration times of 300 and 2000 h (**a**–**d**) and as a function of the swelling ratio for different NMBA concentrations (**e**,**f**). The lines are visual aids.

Figure 11 shows Young’s modulus as a function of the NMBA concentration and swelling ratio at a hydration time of 300 h with 0.1 and 1 wt. % CNC (a and c, respectively) and at a hydration time of 2000 h with 0.1 and 1 wt. % CNC (b and d, respectively). The values are reported in Table 5 and Table 6 at 300 and 2000 h swelling times, respectively. It is evident that, in all cases, Young’s modulus increases with the NMBA concentration, and the values are lower at a hydration time of 2000 h than at that of 300 h; the CNC type does not have an effect, and the variation between the values is indeed the standard deviation for all measurements. Figure 11e,f show Young’s modulus as a function of the swelling ratio at times of 300 and 2000 h for the hydrogels with and without CNCs and with different NMBA concentrations. In both Figure 11e,f Young’s modulus decreases with the NMBA concentration, and it is evident that, for a lower NMBA concentration and a swelling ratio higher than 10, Young’s modulus tends to have the same value with some random variation. The effect of CNC can be observed when comparing the solid and hollow symbols in both graphs; most of the time, Young’s modulus is higher when CNC is 1 wt. %. A statistical analysis is presented in Table 7, and it is evident that, at 300 h, the effects of the NMBA concentration, CNC concentration, and type on Young’s modulus are significantly different, and only the interaction between CNC concentration and type is not significant. At 2000 h, only the NMBA concentration is significantly different when analyzed according to the CNC type.

**Figure 12:**
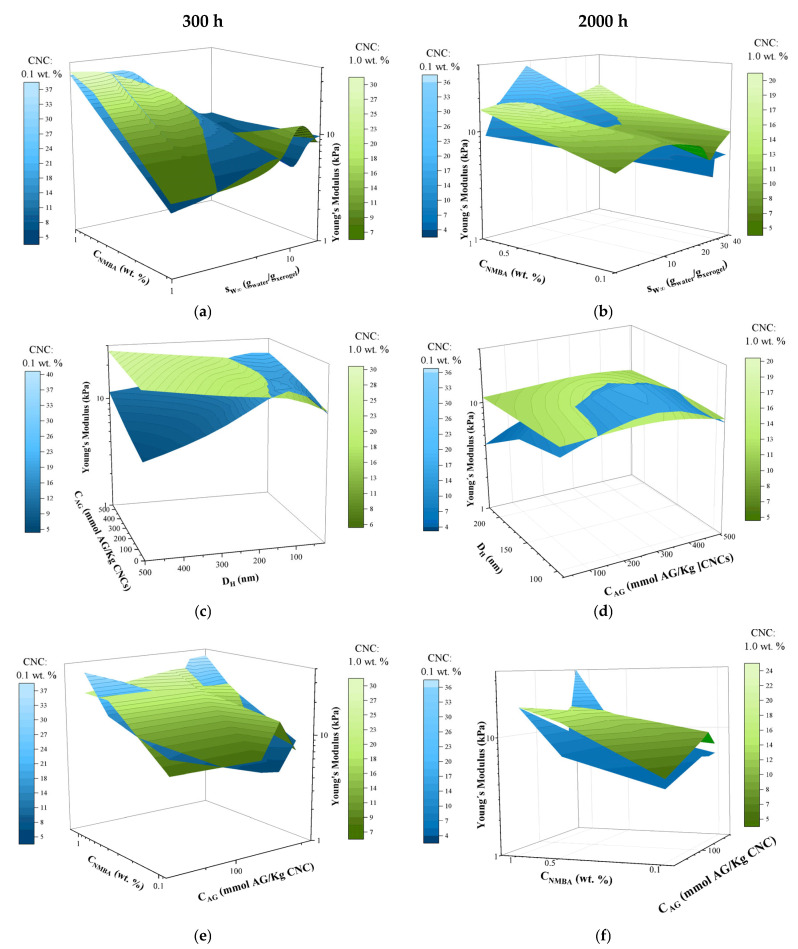
Response surface of Young’s modulus of AAc/AAM hydrogels as a function of the following: NMBA concentration and hydrogel swelling ratio at (**a**) 300 h and (**b**) 2000 h; CNC characteristics (D_H_ and C_AG_) at (**c**) 300 h and (**d**) 2000 h; and NMBA and acid group concentrations at (**e**) 300 and (**f**) 2000 h.

Figure 12 shows the response surface of Young’s modulus as a function of the NMBA concentration and equilibrium swelling degree (S_W∞_) for the hydrogels with 0.1 and 1 wt. % cellulose swollen for (a) 300 h and (b) 2000 h. At 300 h and 0.1 wt. % CNC (Figure 12a), it can be seen that Young’s modulus increases with the NMBA concentration and decreases with the swelling degree. However, at low NMBA concentrations, a slight increase is observed, which is lower than the variation in the results (see Table 5).

It was expected that higher CNC concentrations would cause Young’s modulus to increase, but this was only true for swellings less than 10. For higher S_W∞_, two behaviors occur: at low NMBA concentrations, the reinforcing effect of CNC is enhanced, while at high NMBA concentrations, Young’s modulus decreases drastically. It has been reported that for composite materials, there is a concentration above which the properties are reduced with the concentration of the composite; in this case, the combination of the concentration of the crosslinking agent and the reinforcing material produces the same effect. Crosslinkers modify the hydrogel’s mechanical properties via interactions with polymer networks [128]. Based on a tetrafunctional crosslinker (ethylene dimethacrylate) and poly(ethylene glycol), the system exhibited significant differences in swelling and mechanical properties in terms of their nature and concentration [129]. After 2000 h of swelling, Young’s modulus of the hydrogels shows the same trend (Figure 12b); it can be observed that, regardless of the CNC concentration, the modulus increases with the NMBA concentration. An improvement in the mechanical properties is also observed for 1 wt. % CNC at low crosslinker concentrations and for 0.1 wt. % at high crosslinker concentrations. Adding CNC to PVA hydrogels enhances the mechanical strength by 303% at a deformation of 60% [130]. Additionally, anionic and cationic CNCs form hydrogen bonds, which improve the hydrogel’s stability and strength [131]. Table 5 and Table 6 show Young’s modulus for the hydrogel as a function of the NMBA concentration and CNC source and concentration at swelling times of 300 and 2000 h, respectively. Young’s modulus increases with the NMBA and CNC concentrations and decreases with the swelling time.

Figure 12c,d show the CNCs’ morphological and chemical properties, such as the hydrodynamic radius and concentration of acidic groups, according to Young’s modulus after 300 and 2000 h of swelling. For 1% CNC, it is observed that Young’s modulus generally increases with the CNC length, at both 300 and 2000 h, except for with long lengths and low C_AG_ at 2000 h, as can be seen in Figure 12b. The loss of mechanical properties due to swelling time, i.e., at higher swellings, is determined by comparing 1% CNC at 300 and 2000 h (Figure 12c and Figure 12d, respectively), with this loss being more significant for long CNC lengths and low C_AG_. For 0.1% CNC, the dependence of Young’s modulus on the CNC characteristics becomes more complex; at 300 h, Young’s modulus increases, with respect to that obtained with 1% CNC, at short lengths and high C_AG_. At 2000 h, the loss of properties is evident and more noticeable at the response surface extremes. To determine the effect of the contribution of the crosslinking agent and the acid groups in the CNCs, the response surface is presented in Figure 12e,f for hydration times of 300 and 2000 h, respectively. In both cases, Young’s modulus is higher for hydrogels with 1 wt. %, increases with the NMBA concentration, and exhibits complex behavior with the acid group concentrations.

Finally, this work provides evidence for the reinforcing role of CNCs in the mechanical properties of hydrogels. Some authors have explained this phenomenon from different perspectives according to their studies. Yan et al. (2014) demonstrated the formation of colloidal clusters that create a homogeneously crosslinked network; these clusters enhance the mechanical properties because of dissipated energy during deformation [55]. Conversely, Dellatolas et al. (2023) identified interactions between the CNCs and hydrogel matrix, which increase the polymer density around the CNCs that form fillers in the network, thereby constraining displacement and enhancing stress [132].

## 3. Conclusions

Hydrogels with an acrylic monomer ratio (AAm/AAc) of 50/50 and reinforced with CNCs obtained from various sources were synthesized using a photosensitive initiator. An increase in the N-N methylene bis-acrylamide (NMBA) concentration decreased the swelling capacity. Consequently, the hydrogel presented more resistance to compression, exhibiting a higher Young’s modulus value regardless of the CNC concentration.

The degree of substitution of sulfate groups in the CNCs was determined under various hydrolysis conditions. FTIR was also used to examine the functional groups of the hydrogels. The effect of NMBA manifested in the band at 1271 cm^−1^, while a decrease in the normalized area bands (A/A_1731_) was observed for the wavenumbers of 1455 and 2838 cm^−1^ with the increase in the concentration of acid groups in the cellulose. This behavior suggests that a higher degree of substitution limits the interaction between the OH groups of cellulose and the reaction system, hindering crosslinking reactions. Consequently, a higher concentration of acid groups in the CNCs was associated with a lower degree of hydrogel crosslinking; therefore, these CNCs exhibited a higher swelling capacity than the CNCs with a lower concentration of sulfate groups, resulting in a reduction in the mechanical properties.

Nevertheless, this behavior is more complex, as the mechanical properties depended more on the synergies between the concentrations of the crosslinking agents and CNCs than on the concentration of sulfate groups in the CNCs. At low NMBA concentrations, CNCs improved the mechanical properties. However, at high concentrations, these properties deteriorated, suggesting the presence of percolation conditions due to the interaction between NMBA and CNCs in the hydrogel. Conversely, morphological analysis revealed that the pore size decreased with the increase in the NMBA and CNC concentrations and the incorporation of CNC-generated hydrogels with softer textures. Finally, it was found that the swelling kinetic behavior of the hydrogel fit the second-order Schott equation for times lower than 500 h; for longer times, the swelling behavior was linear, and the mechanical properties decreased with the swelling degree.

## 4. Materials and Methods

### 4.1. Materials

Sulfuric acid (H_2_SO_4_, 99%), sodium hypochlorite (NaClO_2_, 97% purity), sodium hydroxide (NaOH, 97% purity), methanol (CH_3_OH, 99% purity), and hydrogen peroxide (H_2_O_2_, 30% purity) from Golden Bell Reagents (Zapopan, Mexico) were used. Acrylamide AAm (CH_2_=CHCONH_2_, 99%, purity), acrylic acid AAc (CH_2_=CHCOOH 99%, purity), N,N-methilenebisacrylamide NMBA ((CH_2_=CHCONH)_2_CH_2_, 99%, purity), 2,2-dimethoxy-2-phenylacetophenone (C_6_H_5_COC(OCH_3_)_2_C_6_H_5_, 99%, purity), cupriethylenediamine CED (Cu(H_2_NCH_2_NH_2_)_2_(OH)_2_, 1M in H_2_O, 97% purity), and anthraquinone (C_14_H_7_NAO_5_S, 97%, purity) were purchased from Sigma-Aldrich (Toluca, Mexico). Sodium thiosulfate (Na_2_S_2_O_3_, 0.1 N 96% purity) was acquired from Karal Reactivos Analíticos (León, Mexico).

### 4.2. Preparation and Characterization of Soluble-Grade Cellulose Pulp

Huizache wood from Tala, Jalisco, Mexico, was used. The wood was chipped and dried in the environment for 72 h, and then it was sifted and classified. The chips used in this work were those retained in mesh 7. Soluble-grade pulp was obtained using the method reported by Lopez (2017) [133], which consists of pre-hydrolysis, alkaline cooking, and six bleaching steps [15]. For the pre-hydrolysis process, 500 g of chips was mixed with a 0.5% sulfuric acid solution and heated to 160° C for 30 min. The material was then washed and subjected to an alkaline pulping process, with a 24% Na_2_SO_3_/NaOH solution (70/30 *w*/*w* ratio) with 0.1% anthraquinone and 20%V methanol [134]. Cooking was carried out in a Jayme digester (Manufacturer: Scientific and Technical Services at the UPC, Barcelona, Spain), with a 5:1 hydromodule, at 170 °C for 150 min. The resulting pulp was washed and purified for subsequent bleaching using the following bleaching sequence: chlorination–alkaline extraction–chlorine dioxide–hydrogen peroxide–alkaline extraction–hydrogen peroxide (C-E1-D-P1-E2-P2) [135].

The resulting Huizache wood pulp was evaluated by measuring various parameters of interest, such as the Kappa number (TAPPI T 236 om-22) [136], viscosity (TAPPI T 230 om-19) [137], whiteness (TAPPI T 452 om-18) [138], and α, β, and γ-cellulose contents (TAPPI T 203 cm-22) [139].

### 4.3. Obtaining and Characterization of Cellulose Nanocrystals

Before the hydrolysis reaction, the soluble-grade cellulose pulp was pulverized by grinding in a knife mill, equipped with a 200-mesh sieve. The CNCs were obtained via acid hydrolysis, using sulfuric acid at different concentrations, as well as various temperatures and treatment times (see Table 8). The cellulose nanocrystal suspensions were centrifuged, dialyzed, filtered, and stored at 4 °C. The average size of the CNCs and the residual load of strong acid groups were determined in mmol per kg of CNCs.

#### 4.3.1. Determination of the Residual Charge in NCC via Conductance Titration

A conductimetric analysis was performed by placing a conductivity electrode and a pH electrode of a Thermo Scientific Orion Star A215 pH meter (Waltham, MA, USA) inside a three-neck flask. At the same time, a NaOH 0.05 solution was placed in a digital burette. Both devices were connected to a computer in order to record the volume of the added sodium hydroxide and the corresponding pH and conductivity values (mS/Cm). The evaluations were conducted in an inert environment with argon to prevent CO_2_ absorption during the analysis. The conductimetric titrations were carried out in duplicate.

#### 4.3.2. Particle Size Distribution Determined Using Dynamic Light Scattering (DLS)

DLS equipment of the Malvern brand, model Zetasizer Nano S90 (Malvern, Worcestershire, UK), was used. Previously, the CNC samples were sonicated in an Elma brand ultrasound bath (Singen, Germany) for 5 min at room temperature. The obtained CNC suspensions were diluted to 0.2% *v*/*v* and filtered in a 1.6 µm mesh. Then, 200 μL of the suspension was taken and diluted by adding 800 μL of deionized water (18 MΩ-cm). A series of three scans with ten repetitions was conducted for each sample obtained under different hydrolysis conditions.

#### 4.3.3. AFM Morphological Analysis

The cellulose nanoparticle samples were diluted to a concentration of 0.2% *v*/*v* and filtered using a syringe filter with a 25 mm diameter and a 1.6 µm pore size. The suspensions were then sonicated in an ultrasonic bath at a frequency of 20 KHz for 5 min. Finally, 10 µL of the suspensions was deposited on V1-grade mica discs and allowed to dry at room temperature. The CNCs were analyzed using a Park Systems AFM microscope (Park Systems Corp, Suwon, Republic of Korea). The tapping mode technique was used. Amplitude, height, and phase images of 2 × 2 μm and 5 × 5 μm sections of the sample were taken, with a resolution of 512/512 pixels/line. Aluminum-coated silicon microtips, model AC160TS-R3 (from Oxford Instruments, Abingdon, UK), were used for image acquisition.

### 4.4. Hydrogel Synthesis and Characterization

#### 4.4.1. Hydrogel Synthesis

Hydrogels were synthesized via solution polymerization. The monomeric composition of AAc/AAm was 50/50 *w*/*w* and the study variables were as follows: the crosslinking agent concentration (C_NMBA_: 0.1, 0.5, and 1 wt. % as a function of the monomeric phase); the CNC concentration (C_CNC_: 0.1 and 1 wt. % concerning the monomers); and the CNC type, namely, CNCs from Huizache (Hu-Hu-B, Hu-C, and Hu-D), CNCs from agave bagasse, and commercial CNCs from wood (Table 8). The compositions of the hydrogels with respect to the variables are shown in Table 9.

The procedure consisted of mixing 10 g of acrylic acid, 10 g of acrylamide, and NMBA 0.1, 0.5, or 1 % by weight in agreement with the monomeric phase. Samples without CNCs (control) and with 0.1 or 1 wt. % CNC concerning the monomeric phase were prepared. Subsequently, double-distilled water was added to 50 mL of the reaction solution, which was placed in a refrigerator until reaching 3 °C ± 1. Finally, 1 mL of a photoinitiator solution was added (3 g of 2,2-dimethoxy-2-phenylacetophenone in 100 mL of methanol). The reaction solution was poured into a 0.450 L glass semi-infinite plate reactor, which was placed in an isothermal bath at 3 °C. An F15T8-BLB lamp (20W of 127 v), Tecno brand (purchased from Electrica Variedades Guadalajara, Guadalajara, Mexico), was placed 20 cm away from the reaction system; the lamp was rich in radiation with a 366 nm wavelength. Afterward, cylindrical test tubes of 10 mm in diameter and 5 mm in height were stamped. The test tubes were dried and weighed, with the weight recorded as W_0_. Then, polymers were washed by immersing them in double-distilled water and changing the water every 24 h for a week to eliminate the residues of unreacted monomers and polymers not bound to the network. They were left to dry in an oven at 30 °C for 3 days to obtain xerogels, and then they were weighed, with the weight recorded as Wx. The polymer gel fraction (GF) was calculated as follows:

(2) $$GF=\frac{W_{x}}{W_{0}}100$$

where W_x_ is the weight of the dry, insoluble polymer (xerogel) after washing with water, and W_0_ is the initial weight of the polymer.

#### 4.4.2. FTIR Spectroscopy

The samples were dried in an oven at 50 °C for 48 h. Spectra were obtained by using a Perkin Elmer FTIR spectrophotometer, model Spectrum GX (Waltham, MA, USA). A total of 24 scans were taken in the range of 4000 to 400 cm^−1^, and the procedure was performed in duplicate.

#### 4.4.3. Swelling Kinetics

The dried samples were weighed and placed in double-distilled water at 25 °C. Five samples were analyzed for each hydrogel composition, and the average of these five measurements was reported. The samples were weighed at different hydration times. The absorbent paper was used to remove the excess water from the samples’ surfaces. The amount of water absorbed was calculated via the difference in weight between the dry sample and the swollen sample using the following equation:

(3) $$S_{W}=\frac{W_{t}-W_{0}}{W_{0}}$$

where W_t_ is the weight of the hydrogel at time t, and W_0_ is the weight of the xerogel. The experimental results were fitted in agreement with the second-order model proposed by Schott [110], which is commonly used to predict swelling in acrylic hydrogels [4,140]:

(4) $$\frac{{dS}_{W}}{dt}=k{\left(S_{W\infty }-S_{W}\right)}^{2}$$

where S_W_ and S_W∞_ are the swellings at time t and the equilibrium, respectively, and K is a system constant.

By integrating Equation (4), we obtain the following:

(5) $$S_{W}=\frac{k{\;S}_{W}^{2}\;t}{1+k{\;S}_{W}\;t}$$

Equation (5) represents the second-order kinetics in quadratic form; in other words, the swelling rate at any time is directly proportional to the square of the swelling capacity still available, i.e., the solvent uptake that has not yet occurred before reaching the maximum or equilibrium uptake [140].

#### 4.4.4. Scanning Electron Microscopy (SEM)

The xerogels were hydrated for various hydration times, immersed in liquid nitrogen for 3 min for freezing, and immediately fractured. They were freeze-dried using Labconco (Kansas, MO, USA) equipment for 48 h at a temperature of −47 °C and a vacuum of 70 × 10^−3^ Mbar. The xerogels were then re-coated with Au. The samples were observed using a TESCAN electron microscope, model MIRA 3 LMU from Keyence (Osaka, Japan), using an acceleration voltage of 10 kV.

#### 4.4.5. Rheological Characterization of Hydrogels

The Young’s modulus values of the swollen hydrogels were determined at equilibrium, using cylindrical specimens subjected to compression deformation in an AR G2 rheometer (TA Instruments, New Castle, DE, USA); said deformation was carried out at a constant speed of 8 μm/s and 25 °C, with a textured geometry of 25 mm in diameter. The compressive strength, or the capacity of a sample to resist compression loads, was measured by crushing a cylindrical sample according to ASTM-D695-23 [141], where the test sample is placed in a compression instrument, and one of the pistons advances at a constant speed. The maximum compressive strength is equal to the load that causes the breakage of the material divided by the minimum cross-section, as there are many materials that do not break with compression, and the strengths that cause deformation are recorded [142]. From the data obtained (normal force and displacement or gap), the deformation was determined according to δ = (h_0_ − h)/h_0_, where h_0_ is the height of the hydrogel in mm (10.4 ± 3.2), measured individually for each hydrogel, considering h_0_ as the gap when the normal force is different from zero while the geometry descends to compress the hydrogel, and h is the distance that the hydrogel is compressed at a compression rate of 8 μm/s. The compressive strength in Pa was determined by the ratio of the normal force in N and the area of the hydrogel in m^2^. The area of the hydrogel cross-section was determined by measuring the diameter of the cylinder with a Vernier caliper with a precision of 0.05 mm. The Young’s modulus values correspond to the slope of the first linear region (δ < 5 %) of the compressive strength as a function of compression [143]. See Appendix C for an example of Young’s modulus determination.

### 4.5. Statistical Analysis

The residual charge was measured in duplicate, and the average values were reported. The DLS measurement was carried out three times, with ten repetitions each, and the CNC length was obtained by counting around 25 crystals via AFM. Additionally, the swelling kinetic and Young’s modulus values correspond to the average of five measurements (155 data points each). A statistical analysis was performed using OriginLab, with a two-way ANOVA. Factors A, B, and C were the NMBA concentration, CNC concentration, and CNC type, respectively. In the two-way ANOVA, factors A and B were analyzed to determine whether they significantly differed and whether the interaction between them was significant; the values are reported as p_A_, p_B_, and p_AB_. Using the same procedure, factors A and C and factors B and C were combined; the values are reported as p_A_, p_C_, and p_AC_, and p_B_, p_C_, and p_BC_, respectively. In all cases, the significance level used was 0.05.

## Figures and Tables

**Table 1:** The hydrodynamic diameter of CNCs obtained using DLS and acid group concentration.

CNC Source	CNC ID	DLS			AFM	
		D_H_ (nm)	PDI	Type of Curve	L (nm)	[AG] (mmol AG/Kg CNC)
Huizache	Hu-A	142 ± 12	0.32 ± 0.03	Monomodal	180 ± 20	334 ± 32
	Hu-B	89.5 ± 5	0.27 ± 0.03	Bimodal	ND	505 ± 22
	Hu-C	116 ± 22	0.62 ± 0.14	Bimodal	120 ± 50	27 ± 4
	Hu-D	219 ± 40	0.69 ± 0.24	Bimodal	199 ± 93	216 ± 27
Agave bagasse	AB	601				* ND
Commercial Wood	CW	276				42 (0.4 wt. %)

* ND: The measurement was not realized.

**Table 2:** Schott equation parameters for swelling kinetics of AAc/AAm hydrogels as a function of the NMBA and CNC concentrations and CNC type.

CNC Concentration (wt. %)				0.1			1.0		
CNC Source	CNC ID		[NMBA] (wt.%)	0.1	0.5	1	0.1	0.5	1
		Schott Equation Parameters							
Control Sample (Without CNC)		S_W∞_ (g_water_/g_xerogel_)		8.12 ± 1.03	4.79 ± 0.14	4.64 ± 0.31	8.12 ± 1.03	4.79 ± 0.14	4.64 ± 0.31
		K × 10^3^ (h^−1^)		2.9 ± 0.3	5.8 ± 0.27	6.97 ± 0.16	2.9 ± 0.3	5.8 ± 0.27	6.97 ± 0.16
		R^2^		0.9833 ± 0.0033	0.9884 ± 0.0032	0.9870 ± 0.0063	0.9833 ± 0.0033	0.9884 ± 0.0032	0.9870 ± 0.0063
Huizache	Hu-A	S_W∞_ (g_water_/g_xerogel_)		10.2 ± 2.13	3.70± 0.12	2.71 ± 0.13	8.46 ± 1.19	3.77 ± 0.27	2.77 ± 0.14
		K × 10^3^ (h^−1^)		2.1 ± 0.6	8.1 ± 0.5	11.2 ± 2	2.85 ± 0.5	5.95 ± 0.5	10 ± 0.5
		R^2^		0.9868 ± 0.0015	0.9906 ± 0.0010	0.9909 ± 0.0031	0.9908 ± 0.0034	0.9879 ± 0.0026	0.9872 ± 0.0021
	Hu-B	S_W∞_ (g_water_/g_xerogel_)		13.8 ± 0.96	4.45 ± 0.29	2.89 ± 0.07	11.54 ± 0.92	5.24 ± 0.38	2.38 ± 0.25
		K × 10^3^ (h^−1^)		1 ± 0.2	4.2 ± 0.6	17 ± 2.5	1.15 ± 0.14	4.5 ± 0.7	28 ± 2
		R^2^		0.9857 ± 0.0035	0.9900 ± 0.0020	0.9779 ± 0.0023	0.9755 ± 0.0021	0.9924 ± 0.0015	0.9753 ± 0.0043
	Hu-C	S_W∞_ (g_water_/g_xerogel_)		7.79 ± 0.62	3.11 ± 0.10	2.66 ± 0.18	7.61 ± 0.93	3.70 ± 0.22	2.91 ± 0.98
		K × 10^3^ (h^−1^)		1.7 ± 0.3	14 ± 0.9	11.1 ± 3	1.9 ± 0.4	6.5 ± 1	10 ± 1
		R^2^		0.9786 ± 0.0027	0.9844 ± 0.0022	0.9838 ± 0.0098	0.9756 ± 0.0020	0.9782 ± 0.009	0.9846 ± 0.0098
	Hu-D	S_W∞_ (g_water_/g_xerogel_)		7.82 ± 1.95	4.19 ± 0.33	2.52 ± 0.07	17.2 ± 1.4	3.99 ± 0.96	2.79 ± 0.10
		K × 10^3^ (h^−1^)		1.5 ± 0.7	6.4± 0.9	29 ± 4	0.39 ± 0.007	7.1 ± 0.9	21.6 ± 1.9
		R^2^		0.9657 ± 0.0203	0.9788 ± 0.0072	0.9884 ± 0.0016	0.9814 ± 0.0032	0.9802 ± 0.0022	0.9898 ± 0.0020
Agave bagasse	AB	S_W∞_ (g_water_/g_xerogel_)			5.95 ± 0.41			4.54 ± 0.35	
		K × 10^3^ (h^−1^)			4 ± 0.4			6.5 ± 0.7	
		R^2^			0.9724 ± 0.0076			0.9900 ± 0.0032	
Commercial Wood	CW	S_W∞_ (g_water_/g_xerogel_)			4.35 ± 0.33			3.61 ± 0.31	
		K × 10^3^ (h^−1^)			4.1 ± 0.09			9.1 ± 1	
		R^2^			0.9913 ± 0.0022			0.9911 ± 0.0041	

**Table 3:** Statistical analysis of hydrogel swelling kinetics at 300 and 2000 h.

Factors		Schott Model (t < 300 min)		Linear Regression 350 < t < 1700	
		K	S_W∞_	b	m
A, B	p_A_	p < 0.05	p < 0.05	p < 0.05	p < 0.05
	p_B_	p > 0.05	p > 0.05	p < 0.05	p < 0.05
	p_AB_	p < 0.05	p < 0.05	p < 0.05	p < 0.05
A, C	p_A_	p > 0.05	p > 0.05	p < 0.05	p > 0.05
	p_C_	p < 0.05	p < 0.05	p < 0.05	p < 0.05
	p_AC_	p < 0.05	p < 0.05	p < 0.05	p < 0.05
B, C	p_B_	p > 0.05	p > 0.05	p < 0.05	p > 0.05
	p_C_	p > 0.05	p > 0.05	p > 0.05	p > 0.05
	p_BC_	p > 0.05	p > 0.05	p > 0.05	p > 0.05

Factor A: NMBA concentration. Factor B: CNC concentration. Factor C: CNC type. At p < 0.05, factors A, B, or C are significantly different or the interaction between two factors is significant. At p > 0.05, factors A, B, or C are not significantly different or the interaction between two factors is not significant.

**Table 4:** Line equation parameters for swelling kinetics of AAc/AAm hydrogels as a function of the NMBA and CNC concentrations and CNC type.

CNC Concentration (wt. %)				0.1			1.0		
CNC Source	CNC ID		[NMBA] (wt.%)	0.1	0.5	1	0.1	0.5	1
		Slope Lineal Equation							
Control Sample (Without CNC)		b		13.6 ± 0.7	4.72 ± 0.24	4.19 ± 0.45	13.6 ± 0.7	4.72 ± 0.24	4.19 ± 0.45
		m		0.0139 ± 9.9 × 10^−4^	0.0032 ± 2.6 × 10^−4^	0.0032 ± 2.6 × 10^−4^	0.0139 ± 9.9 × 10^−4^	0.0032 ± 2.6 × 10^−4^	0.0032 ± 2.6 × 10^−4^
		R^2^		0.9830 ± 0.032	0.9696 ± 0.049	0.9937 ±0.0031	0.9830 ± 0.0032	0.9696 ± 0.049	0.9937 ± 0.0031
Huizache	Hu-A	b		10.2 ± 2.2	3.24 ± 0.14	2.14 ± 0.08	8.4 ± 1.5	2.78± 0.32	2.07 ± 0.14
		m		0.0104 ± 5.3 × 10^−4^	0.0034 ± 1.9 × 10^−4^	0.0013 ± 2.5 × 10^−4^	0.0114 ± 1.2 × 10^−3^	0.0024 ± 1.7 × 10^−4^	0.0016 ± 1.3 × 10^−4^
		R^2^		0.9881 ± 0.0077	0.9937 ± 0.0025	0.9287 ± 0.1197	0.9925 ± 0.0051	0.9803 ± 0.03	0.9897 ± 0.003
	Hu-B	b		9.70 ± 0.56	4.11 ± 0.27	2.24 ± 0.07	7.64 ± 0.81	2.95 ± 0.21	1.83 ± 0.29
		m		0.0125 ± 4.6 × 10^−4^	0.0032 ± 2.1 × 10^−4^	0.0104 ± 5.3 × 10^−4^	0.0105 ± 4.7 × 10^−4^	0.0032 ± 1.2 × 10^−4^	0.0015 ± 3.3 × 10^−4^
		R^2^		0.9853 ± 0.011	0.9938 ± 0.0027	0.9881 ± 0.0077	0.9964 ± 0.0009	0.9865 ± 0.0072	0.9765 ± 0.02
	Hu-C	b		5.19 ± 0.57	1.99 ± 0.15	1.81 ± 0.18	6.15 ± 1.24	2.63 ± 0.12	1.94 ± 0.19
		m		0.0109 ± 8.5 × 10^−4^	0.0032 ± 3.4 × 10^−4^	0.0019 ± 2.1 × 10^−4^	0.009 ± 7.2 × 10^−4^	0.0029 ± 4.7 × 10^−4^	0.0022 ± 3.9 × 10^−4^
		R^2^		0.9886 ± 0.0047	0.9917 ± 0.006	0.9881 ± 0.006	0.9963 ± 0.0025	0.9913 ± 0.0034	0.9870 ± 0.006
	Hu-D	b		9.21 ± 0.81	3.05 ± 0.13	2.15 ± 0.04	4.07 ± 0.78	2.66 ± 0.08	2.36 ± 0.08
		m		0.0137 ± 9.2 × 10^−4^	0.0034 ± 1.6 × 10^−4^	0.00104 ± 8.9 × 10^−5^	0.0087 ± 1.6 × 10^−3^	0.0035 ± 1.6 × 10^−4^	0.00108 ± 1.4 × 10^−4^
		R^2^		0.9987 ± 0.0006	0.9900 ± 0.0016	0.9819 ± 0.0044	0.9982 ± 0.0013	0.9924 ± 0.0041	0.9814 ± 0.005
Agave bagasse	AB	b			2.94 ± 0.20			2.64 ± 0.30	
		m			0.0028 ± 3.5 × 10^−4^			0.0022 ± 3.4 × 10^−4^	
		R^2^			0.9822 ± 0.006			0.9831 ± 0.01	
Commercial Wood	CW	b			4.56 ± 0.04			3.26 ± 0.29	
		m			0.0036 ± 3.3 × 10^−4^			0.0027 ± 1.1 × 10^−4^	
		R^2^			0.9810 ± 0.02			0.9844 ± 0.004	

For $S_{W}=b+mt$, m is in g_water_/g_xerogel_ h^−1^ and the m value is 0.005 ± 0.004.

**Table 5:** Young’s modulus (kPa) of AAc/AAm hydrogels as a function of CNC type and concentration and NMBA concentration after 300 h of swelling.

C_CNC_ (wt. %)		0.1			1		
CNC Type	C_NMBA_ (wt. %)	0.1	0.5	1	0.1	0.5	1
Control		9.7 ± 1.8	10.5 ± 6.2	17.5 ± 7.2	9.7 ± 1.8	10.5 ± 6.2	17.5 ± 7.2
Huizache	Hu-A	4.8 ±1.8	13.1 ± 5.6	35.3 ± 7.3	6.6 ± 1.5	21.3 ± 1.7	28.7 ± 12.2
	Hu-B	8.8 ± 2.5	19.8 ± 3.6	36.6 ±8.7	13.9 ± 2.7	15.3 ± 4.1	29.1 ± 4.3
	Hu-C	9.5 ± 5.1	15.3 ± 7.6	33.7 ± 16.4	6.8 ± 1.4	21.4 ± 11.3	21.8 ± 4.0
	Hu-D	5.1 ± 1.7	15.8 ± 4.5	16.5 ± 1.9	7.2 ± 2.3	20.4 ± 7.6	29.6 ± 9.0
Agave bagasse			14.6 ± 8.5			17.4 ± 4.0	
Comm. Wood			19.8 ± 9.7			25.6 ± 19.6	

**Table 6:** Young’s modulus (kPa) of AAc/AAm hydrogels as a function of CNC type and concentration and NMBA concentration after 2000 h of swelling.

C_CNC_ (wt. %)		0.1			1		
CNC Type	C_NMBA_ (wt. %)	0.1	0.5	1	0.1	0.5	1
Control		5.8 ± 1.2	8.6 ± 2.4	19.3 ± 9.5	5.8 ± 1.2	8.6 ± 2.4	19.3 ± 9.5
Huizache	Hu-A	5.7 ± 1.6	10.3 ± 2.3	3.6 ± 3.0	9.4 ± 2.9	13.7 ± 1.4	18.4 ± 1.3
	Hu-B	5.9 ± 0.2	6.3 ± 4.3	17.2 ± 8.6	7.2 ± 1.7	8.8 ± 1.5	15.7 ± 4.4
	Hu-C	3.9 ± 0.6	6.9 ± 3.7	17.8 ± 2.7	4.7 ± 0.6	13.1 ± 1.6	17.7 ± 2.1
	Hu-D	5.9 ± 0.7	6.7 ± 2.9	9.0 ± 4.1	8.1 ± 1.6	12.3 ± 1.2	15.2 ± 5.9
Agave bagasse			11.0 ± 4.8			15.3 ± 3.3	
Comm. Wood			16.1 ± 2.3			23.6 ± 5.8	

**Table 7:** Statistical analysis of mechanical properties of hydrogels at 300 and 2000 h.

		Y (300 h)	Y (2000 h)
A, B	p_A_	p < 0.05	p > 0.05
	p_B_	p < 0.05	p > 0.05
	p_AB_	p < 0.05	p > 0.05
A, C	p_A_	p < 0.05	p < 0.05
	p_C_	p < 0.05	p > 0.05
	p_AC_	p < 0.05	p > 0.05
B, C	p_B_	p < 0.05	p > 0.05
	p_C_	p > 0.05	p > 0.05
	p_BC_	p > 0.05	p > 0.05

**Table 8:** Experimental conditions for obtaining nanocrystals.

CNC Source	CNC ID	[H_2_SO_4_]	T (°C)	t (min)	Filter Size (μm)
Huizache	Hu-A	62.5	50	55	1.6
	Hu-B	65	55	65	1.6
	Hu-C	60	55	65	1.6
	Hu-D	60	55	45	1.6
Agave bagasse	AB	63.5	44	130	-
Comm. Wood	CW	64	-	-	-

**Table 9:** NMBA and CNC concentrations of hydrogels for the different CNC types.

CNC Type	[NMBA] (wt. %)	[CNC] (wt. %)
Control Sample	0.1, 0.5 and 1	0
Hu-A	0.1, 0.5 and 1	0.1 and 1
Hu-B	0.1, 0.5 and 1	0.1 and 1
Hu-C	0.1, 0.5 and 1	0.1 and 1
Hu-D	0.1, 0.5 and 1	0.1 and 1
AB	0.5	0.1 and 1
CW	0.5	0.1 and 1

**Figure A2:**
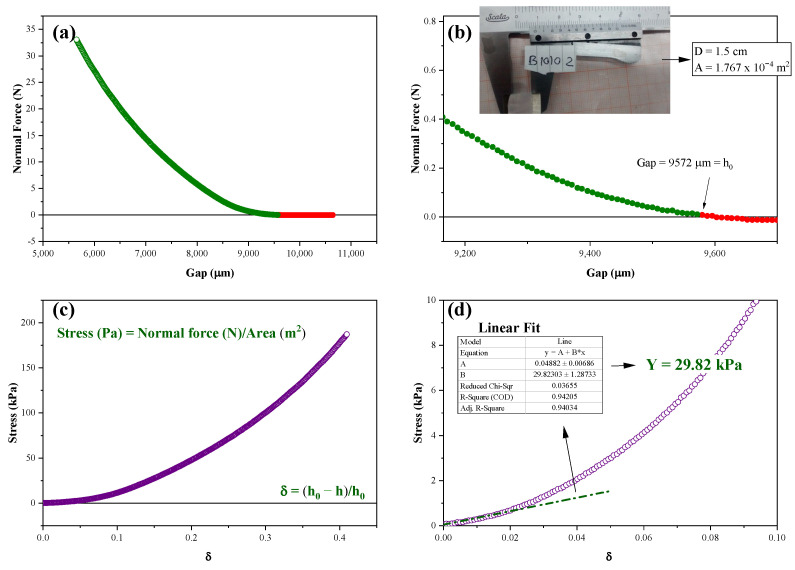
(**a**) CompressionP test for hydrogel with 1 wt. % NMBA and 1 wt. % Hu-B CNC. (**b**) Magnification of the rheogram in (**a**) to obtain the gap when the normal force is different from zero; this gap corresponds to the hydrogel height (h_0_); the data in red are negligible, and for calculus, only data in green are considered; the picture shows the measurement of the hydrogel diameter with a Vernier caliper, which was used to obtain the hydrogel surface area. (**c**) The stress–strain curve was obtained from the data shown in (**a**). The normal force was divided by the hydrogel surface area, and the deformation was obtained by the ratio between the vertical scroll and the hydrogel height (h_0_). (**d**) Young’s modulus was obtained by the linear fit of the first lineal region of the stress–strain curve at very low deformation, the inset shows the fit results, where x and y are deformation and stress, respectively, and slope B corresponds to Young’s modulus.

## Data Availability

The data presented in this study are available upon request from the corresponding author.

## Appendix A. FTIR Analysis

**Table A1:** The FTIR peak analysis, center peak wave number, and area percentage average for the control and hydrogel composite samples obtained from the spectra deconvolution, as well as the percentage of area variation of each and the corresponding functional group associated with the band.

Wavenumber (cm^−1^)	FTIR % A Control Samples	Variation (%)	FTIR % A Composite Hydrogels	Variation (%)	Type of Vibration of Functional Group			Reference
474 ± 5	0.08 ± 0.04	43	0.09 ± 0.07	79	C-C	Deformation	AAm	[100]
803 ± 3	1.09 ± 0.14	13	1.21 ± 0.52	44	CH_2_	Wagging	AAc	[4,144]
					C-H	Wagging	AAm	[100]
960 ± 0.8	1.03 ± 0.03	3	0.91 ± 0.65	71	OH	Deformation	AAc	[98]
1038 ± 6	2.10 ± 0.42	20	1.72 ± 0.28	16	C-O		CNC	[104,105,106]
					CH_2_	Deformation	AAm	[100]
1118 ± 2	2.80 ± 0.44	16	2.37 ± 0.23	10	-CO	Stretching	AAc	[145]
					CC	Asym stretching	AAm	[100]
1169 ± 1.4	3.41 ± 0.45	13	3.24 ± 0.64	17	C-O-C		CNC	[104,105,106]
					C-O	Stretching	AAc	[4]
					C-C	Stretching		[100]
1271 ± 2.6	3.07 ± 0.21	7	2.80 ± 0.21	8	OH	Deformation	AAc	[4,108,145]
1340 ± 0.7	0.05 ± 0.03	53	0.47 ± 0.17	35	CH_2_	Wagging	AAc, AAm, NMBA	[100]
1354 ± 6	1.73 ± 0.22	13	1.20 ± 0.23	19	N-H, C-N with 670	Stretching and flexion	AAm	[4]
					NH_2_	Scissoring	AAm	[100]
1422 ± 3	1.99 ± 0.25	12	1.68 ± 0.20	12	C-O, O-H	C-O stretching and O-H flexion	AAc, NMBA	[98,144]
					C-O, O-H	Flexion	AAm	[4]
					n-crystal		CNC	[103]
1455 ± 2	2.17 ± 0.15	7	2.14 ± 0.19	9	COO-		AAm	
					CH_2_	Asym stretching	AAc	[4,144,145]
					CH_2_	Asym stretching	AAm	[100]
1588 ± 5	4.69 ± 0.32	7	3.35 ± 0.95	29	NH_2_	Deformation	AAm (II)	[4,99,100,101]
1688 ± 21	3.06 ± 0.45	15	4.52 ± 1.55	34	C=O	Stretching	AAm (I)	[4,99,101]
1731 ± 4	10.26± 0.06	0.6	9.23 ± 0.61	6.6	C=O	Stretching	AAc, NMBA	[4]
2182 ± 1.6	0.19 ± 0.31	167	0.24 ± 0.45	188	C-N	Stretching	AAm	[100]
2537 ± 0	9.46 ± 2.50	26	7.63 ± 2.68	35	N-H	Stretching	AAm	[98]
					O-H	Stretching	AAc	[98]
2643 ± 16	5.98 ± 0.79	13	5.54 ± 0.36	6	N-H	Stretching	AAm	[98]
					O-H	Stretching	AAc	[98]
2838 ± 4	7.78 ± 0.82	10.6	6.48 ± 0.79	12	CH_2_	Asym stretching	AAm	[98]
2883 ± 8	2.68 ± 1.00	37	2.08 ± 0.50	24	CH	Sym Stretching	AAm,	[100])
2942 ± 16	5.11 ± 1.03	20	5.31 ± 0.87	16	CH_2_	Asym stretching	AAm	[100,144]
3019 ± 4	6.87 ± 0.44	6	2.91 ± 2.92	100	CH_2_	Sym stretching	AAm	[100]
3170 ± 0	5.70 ± 0.23	4	6.30 ± 0.63	11	OH	Stretching	AAc	[144]
					NH_2_	Sym stretching	AAm	[4,100]
3326 ± 8	1.73 ± 0.57	33	5.6 ± 3.12	56	OH		AAc, CNC	[98,104,105,106]
					NH_2_	Asym stretching	AAm	[100]
					N-H	Stretching	AAm	[4]
3445 ± 16	10.42 ± 6.8	65	0.08 ± 0.13	153	O-H, N-H		AAc, AAm	[107]
3650 ± 2.5	4.73 ± 1.88	40	4.52 ± 1.49	33	O-H	Stretching	AAc	[98]

## Appendix B. Hydrogel Swelling Kinetic Images

Table A2 shows photographs of the hydrogel kinetics as a function of the hydration time, NMBA concentration, and CNC concentration for different CNC types.

**Table A2:** Photographs of hydrogels at different swollen times as a function of CNC type and CNC and NMBA concentration.

CNCType	C_NMBA_	C_CNC_	Swelling Time (h)				
	(wt. %)		0	3	24	72	144
Control	0.1	0					
	0.5	0					
	1	0					
Hu-A	1	1					
Hu-B	0.1	0.1					
Hu-C	1	0.1					
Hu-D	0.1	1					
AB	0.5	1					
CW	0.5	0.1					
